# Inhibition of N‐Terminal Acetyltransferase C Mitigates Endoplasmic Reticulum Stress–Mediated Muscle Atrophy in Cancer Cachexia

**DOI:** 10.1002/jcsm.70249

**Published:** 2026-03-19

**Authors:** Yusaku Kaneko, Tomohiro Hino, Shunta Taminishi, Yayoi Matoba, Daisuke Motooka, Atsushi Hoshino, Satoaki Matoba

**Affiliations:** ^1^ Department of Cardiovascular Medicine, Graduate School of Medical Science Kyoto Prefectural University of Medicine Kyoto Japan; ^2^ Department of Nephrology, Graduate School of Medical Science Kyoto Prefectural University of Medicine Kyoto Japan; ^3^ Department of Infection Metagenomics, Research Institute for Microbial Diseases Osaka University Osaka Japan; ^4^ Integrated Frontier Research for Medical Science Division, Institute for Open and Transdisciplinary Research Initiatives (OTRI) Osaka University Osaka Japan

**Keywords:** cachexia, ER stress, muscle atrophy, N‐terminal acetyltransferase C

## Abstract

**Background:**

Cancer cachexia is a complex syndrome marked by weight loss and muscle wasting, significantly impacting patient quality of life and survival. Mechanistically, it is characterized by suppressed protein synthesis and enhanced muscle catabolism, with the role of endoplasmic reticulum (ER) stress and unfolded protein response (UPR) becoming increasingly evident. This study aimed to explore ER stress–tolerant factors in muscle wasting and evaluate their potential to prevent muscle loss in cancer cachexia.

**Methods:**

A genome‐wide CRISPR screening was conducted in the context of ER stress–mediated growth inhibition of C2C12 myoblasts. The candidate genes resistant to ER stress were further evaluated in C2C12 myotubes treated with conditioned medium of Lewis lung adenocarcinoma (LLC) cells. Twelve‐week‐old male mice were administered LLC cells and shRNA against Naa35 via adeno‐associated virus. Four weeks later, tibialis anterior (TA) muscles were analysed for muscle mass, grip strength and molecular changes with quantitative polymerase chain reaction, western blotting and histological analysis.

**Results:**

CRISPR screening identified Naa35, Naa38 and Naa30, all three components of N‐terminal acetyltransferase C, as key molecules for resistance to ER stress. The atrophic muscles of mice bearing LLC demonstrated an elevation of UPR, as well as 1.64‐fold upregulation of Naa35 protein (*p* = 0.0072). Among the three branches of the UPR, an ATF6 inhibitor, AEBSF, abolished upregulation of Naa35, Naa38 and Naa30, and an ATF6 activator, AA147, induced Naa35 expression in a dose‐dependent manner (*p* < 0.001). In cells treated with LLC conditioned medium, Naa35 knockdown reduced the amount of cathepsin K (CTSK) protein, which subsequently resulted in the CTSK‐mediated proteolysis of insulin receptor substrate 1. In LLC‐bearing mice, Naa35 knockdown led to a 65.4% reduction in CTSK protein expression (*p* < 0.001) and preservation of the phosphorylation levels of protein kinase B (*p* < 0.0324) and anabolic‐related S6 kinase (*p* < 0.0375). Concurrently, the expression of catabolism‐related genes was repressed (MuRF1, *p* < 0.0015; MAFbx1, *p* < 0.0265). These alterations were associated with the restoration of TA muscle mass (2.52 ± 0.19 vs. 3.72 ± 0.45 mg/g, *p* = 0.0004), fibre area (1741 ± 992 vs. 2099 ± 1264 mm^2^, *p* < 0.0001), grip strength in all four limbs (0.0328 ± 0.0076 vs. 0.0506 ± 0.0130 N/g, *p* = 0.0295) and wire mesh hanging time (496 ± 331 vs. 1038 ± 370 s, *p* = 0.0406).

**Conclusions:**

Inhibition of N‐terminal acetyltransferase C prevents ER stress–induced muscle wasting via the downregulation of CTSK and subsequent activation of the anabolic pathway. This suggests that N‐terminal acetyltransferase C is a potential therapeutic target for combating muscle wasting in cancer cachexia.

## 1 Introduction

Cancer cachexia is a complex and multifaceted syndrome characterized by severe weight loss, muscle wasting and systemic inflammation, negatively affecting the quality of life and survival rates of cancer patients. This syndrome is metabolically associated with the inhibition of protein synthesis and enhancement of muscle catabolism [1]. However, the underlying mechanisms driving this hypercatabolism remain incompletely understood. Recent studies have highlighted the activation of endoplasmic reticulum (ER) stress–induced unfolded protein response (UPR) in skeletal muscle of animal models [2] and muscle biopsies of cachectic cancer patients [3]. The precise role of ER stress and how each arm of the UPR contributes to regulating muscle wasting is just beginning to be understood. ER stress is a phenomenon that occurs when the ER's protein‐folding capacity is exceeded, resulting in the activation of the UPR. This response is designed to restore ER homeostasis; however, it can also trigger cellular pathways towards muscle degradation and systemic inflammation [4]. The interplay between ER stress and cancer cachexia is complex and involves multiple signalling pathways, including the PERK, IRE1α and ATF6 pathways [3]. These pathways can result in the activation of pro‐apoptotic and pro‐inflammatory signals, thereby exacerbating muscle wasting and metabolic dysfunction [5]. For instance, PERK activation can result in the phosphorylation of eIF2α and consequent ATF4 nuclear translocation, which ultimately leads to a reduction in protein synthesis and the promotion of muscle atrophy [6]. Similarly, IRE1 and ATF6 pathways can induce the production of pro‐inflammatory cytokines, thereby contributing to the cachectic phenotype [7]. Further understanding the detailed relationship between ER stress and cancer cachexia is imperative for the development of targeted therapeutic strategies that can prevent muscle loss and improve patient outcomes.

N‐terminal acetylation (Nt‐acetylation) is one of the most prevalent covalent protein modifications, affecting 50%–70% of the yeast and 80%–90% of the human proteome [8]. N‐terminal acetyltransferases (NATs) facilitate the transfer of an acetyl group to the N‐terminus of target proteins, thereby modifying their electrostatic properties. This modification occurs in ribosome‐associated co‐translational and post‐translational manners and affects a number of protein properties, including folding, interaction, localization and proteasomal degradation [9, 10]. Aberrant Nt‐acetylation is implicated in several diseases, including cancers and developmental disorders [11]. All eukaryotes contain five conserved NATs, designated NatA to NatE. Additionally, higher eukaryotes express NatF to NatH [12]. NatC is a heterotrimeric complex comprising a catalytic subunit, Naa30, and two auxiliary subunits, Naa35 and Naa38 [12]. NatC engages in co‐translational actions on the peptide substrate whose N‐terminus begins with methionine followed by a hydrophobic or amphipathic amino acid (ML−, MF−, MI− and MW−) [13]. This Nt‐acetylation with NatC increases protein stability [14] and regulates a wide range of biological processes, including viral particle assembly, maintenance of mitochondrial integrity and apoptosis [9].

To comprehensively understand the role of ER stress in muscle wasting, we conducted a genome‐wide CRISPR library screening in the context of tunicamycin‐mediated ER stress for the growth of C2C12 myoblasts. Knockout of each component of the NatC enzyme—Naa35, Naa38 and Naa30—showed resistance to cell growth inhibition. Consistent with this result, deletion of NatC attenuated myotube atrophy in tunicamycin treatment, as well as in the conditioned medium of Lewis lung adenocarcinoma. Mechanistically, NatC was upregulated via the ATF6 pathway in both the context of ER stress and the cancer cachexia model. NatC stabilized cathepsin K (CTSK) and further modulated the mammalian target of rapamycin (mTOR)–mediated protein homeostasis, where CTSK induced insulin receptor substrate 1 (IRS1) degradation and negatively regulated protein kinase B (Akt). In a mouse model of cancer cachexia, intramuscular injection of AAV9 carrying shRNA against Naa35 counteracted the reduction of tibialis anterior (TA) mass and its functional decline. These findings highlight the role of NatC in the ER stress–mediated muscle wasting and the therapeutic potential of NatC inhibition as a means to prevent muscle wasting in cancer cachexia.

## 2 Materials and Methods

### 2.1 Cells

C2C12 myoblasts (RIKEN BioResource Center, Tsukuba, Japan; RCB0987), Lenti‐X 293T cells (Takara Bio Inc., Shiga, Japan; Cat# 632180), Neuro2A cells (RIKEN BioResource Center, Tsukuba, Japan; RCB2630), Lewis lung carcinoma (LLC) cells (JCRB Cell Bank, Osaka, Japan; JCRB1348) and C26 colon carcinoma cells (ATCC, Manassas, VA, USA; Cat# CRL‐2638) were cultured at 37°C with 5% CO_2_ in Dulbecco's Modified Eagle's Medium (DMEM; Fujifilm Wako Pure Chemical Corporation, Osaka, Japan; Cat# 044‐29765) containing 10% fetal bovine serum (Thermo Fisher Scientific, Waltham, MA, USA) and penicillin–streptomycin (100 U/mL; Thermo Fisher Scientific, Waltham, MA, USA). No commonly misidentified cell line was used in this study. All the cell lines were routinely tested negative for mycoplasma contamination. To establish an ER stress model, C2C12 myoblasts and myotubes were treated with tunicamycin (TM, 160 ng/mL; Fujifilm Wako Pure Chemical Corporation, Osaka, Japan; Cat# 202‐08241) or thapsigargin (TG, 1 μM or 4 nM; Fujifilm Wako Pure Chemical Corporation, Osaka, Japan; Cat# 209‐17281). GSK2606414 (1 μM; Fujifilm Wako Pure Chemical Corporation, Osaka, Japan; Cat# 5107/10) was used as a PERK kinase inhibitor, Kira6 (3 μM; Selleck Chemicals, Houston, TX, USA; Cat# S8658) as a Type II IRE1 kinase inhibitor, AEBSF (400 μM; Thermo Fisher Scientific, Waltham, MA, USA; Cat# 78431) as an ATF6 signalling inhibitor. AA147 (Tocris Bioscience, Bristol, UK; Cat# 6759) was used as an ATF6 activator. For myogenic differentiation, C2C12 cells were grown to approximately 80%–90% confluence, and the culture medium was replaced with differentiation medium consisting of DMEM supplemented with 2% horse serum (Gibco, Thermo Fisher Scientific, Waltham, MA, USA). The differentiation medium was refreshed every 48 h, and myotube formation was allowed to proceed for 4 days before subsequent experiments. Dexamethasone (10 μM; Sigma‐Aldrich, St. Louis, MO, USA; Cat# D4902) was used for glucocorticoid‐induced atrophy experiments.

### 2.2 Mice

C57BL/6J mice (male, 12 weeks old) were purchased from CLEA Japan Inc. (Tokyo, Japan). LLC cells were transplanted under the skin of the back of 12‐week‐old mice to establish a mouse model for tumour transplantation. Transplantation was performed by subcutaneous injection of 2.5 × 10^6^ LLC cells suspended in 100 μL of ice‐cold Hank's Balanced Salt Solution (HBSS; Thermo Fisher Scientific, Waltham, MA, USA). Simultaneously with tumour transplantation, the anterior tibialis muscle was injected intramuscularly with shRNA‐scramble or shRNA‐Naa35 using adeno‐associated virus (AAV9, serotype 9). AAV9 vectors were purchased from VectorBuilder Inc. (Chicago, IL, USA; Order ID: VB211105‐1022zyc). Hamilton syringes were used for injections and 1.0 × 10^10^ genome copies (GC) per limb were injected. Mice were housed in a pathogen‐free specific animal facility on a 13:11 h light–dark cycle. Mice were given water and food *ad libitum*. Each evaluation of the mice was performed after 4 weeks. In compliance with UKCCR guidelines, mice were euthanized as a humane endpoint if tumour diameter exceeded 17 mm or tumour weight exceeded 10% of body weight. All mouse experiments were approved by the Animal Care and Use Committee of the Kyoto Prefectural University of Medicine (M2021‐565, M2022‐116, M2023‐104). Two independent cohorts of mice were used in this study. In the first experiment (initially control *n* = 8, cachexia *n* = 8), two cachectic mice died during the final week due to tumour progression, leaving control *n* = 8 and cachexia *n* = 6 at endpoint. Because the TA muscle is small, the remaining tissue after histological processing did not yield sufficient protein or RNA for molecular analyses. Therefore, a second cohort (control *n* = 4, cachexia *n* = 4) was generated under the same conditions. One cachectic mouse died during the experiment, leaving *n* = 3 at endpoint. Samples from this second cohort provided sufficient material for molecular and histological analyses and were used for these assays.

### 2.3 LLC and C26 Conditioned Medium

LLC cells and C26 cells were seeded with growth medium. After plating for 2 days, additional growth medium was added. The LLC cell cultures contained a heterogeneous mixture of adherent and floating cells. After 4 days, the conditioned medium was centrifuged at 800 rpm for 5 min, filtered through a 0.45‐μm hydrophilic polyvinylidene fluoride (PVDF) filter (Merck Millipore, Cork, Ireland), and stored at −80°C for later use. For myotube treatment, CM was diluted to 50% with fresh differentiation medium. Similarly, conditioned medium from C26 cells (C26‐CM) was prepared and applied to C2C12 myotubes to induce cachectic atrophy.

### 2.4 Immunofluorescence and Analysis of Myotube Size

Two days after treatment of myotube cells with TM (160 ng/mL) or 50% conditioned medium of LLC (LLC‐CM) or DEX (10 μM), myotubes were fixed with 4% paraformaldehyde phosphate buffer Solution (Fujifilm Wako Pure Chemical Corporation, Osaka, Japan; Cat# 163‐20145) and permeabilized with 0.1% Triton X‐100 (Sigma‐Aldrich, St. Louis, MO, USA; Cat# T9284). The cells were blocked with normal donkey serum (Jackson ImmunoResearch, West Grove, PA, USA; Cat# 017‐000‐121) and incubated overnight at 4°C in blocking solution with Anti‐desmin antibody (Abcam, Cambridge, UK; Cat# ab15200; 1:500). The next day, the cells were washed with Phosphate‐buffered saline (PBS; Fujifilm Wako Pure Chemical Corporation, Osaka, Japan; Cat# 165‐23555) and incubated in Anti‐rabbit IgG (H + L), Alexa Fluor 488 (Thermo Fisher Scientific, Waltham, MA, USA; Cat# A11034; 1:500) and photographed under a fluorescence microscope (BZ‐X810; Keyence Corporation, Osaka, Japan).

Images were captured, and the diameter of the myotubes was measured with ImageJ software (National Institutes of Health, Bethesda, MD, USA). The diameter was measured in 150 myotubes selected randomly within each well.

### 2.5 Plasmid

Individual gRNAs were cloned into lentiCRISPR v2 (Addgene, Watertown, MA, USA; plasmid #52961) or lentiGuide‐Puro (Addgene, Watertown, MA, USA; plasmid #52963), and cDNAs were cloned into pLenti (Addgene, Watertown, MA, USA; plasmid #22255) or pMSCV (Takara Bio Inc., Shiga, Japan). Drug‐resistance cassettes in lentiGuide and pLenti vectors were replaced with blasticidin S, neomycin, or puromycin resistance genes. Lentiviral packaging was performed using pMD2.G (Addgene, Watertown, MA, USA; plasmid #12259) and psPAX2 (Addgene, Watertown, MA, USA; plasmid #12260).

### 2.6 Virus Production

To produce viruses, six‐well plates of 70% confluent Lenti‐X 293T cells were transfected with 1.5 μg of transfer vector, 0.5 μg pMD2.G and 1.0 μg of psPAX2 for lentivirus using FuGENE HD Transfection Reagent (Promega, Madison, WI, USA; Cat# E2311) according to the manufacturer's instructions. Supernatant was collected after 48 h and frozen at −80°C.

### 2.7 CRISPR Screening

Cas9‐expressing C2C12 myoblasts were infected with validated lentiviral particles generated from a whole‐genome CRISPR Brie library (Addgene, Watertown, MA, USA; plasmid #73632). After 24 h, infected cells were treated with 5 μg/mL puromycin dihydrochloride (Thermo Fisher Scientific, Waltham, MA, USA; Cat# A1113803) for 2 days. Cells were cultured for further 10 days. The cells expressing the library of gene‐specific gRNAs were then treated with or without tunicamycin (TM, 160 ng/mL) for 2 weeks, followed by genomic DNA isolation and deep sequencing.

### 2.8 Deletion of Target Genes Using CRISPR‐Cas9 System

The expression vector for guide RNA expression was generated using the LentiGuide‐Puro plasmid (Addgene, Watertown, MA, USA; plasmid #52963). These plasmids were transfected into cells expressing Cas9.

### 2.9 FACS Analysis to Evaluate Cell Growth

The effect of individual gRNAs was assessed using a co‐culture system with control cells. Target gRNA‐transduced cells were labelled with GFP, whereas non‐targeting control (NTC) gRNA‐transduced cells were labelled with mCherry. These populations were co‐cultured under TM treatment (160 ng/mL) for 7 days. The relative population ratios were quantified using the Attune NxT Flow Cytometer (Thermo Fisher Scientific, Waltham, MA, USA) to evaluate the impact of each gRNA.

$$\begin{array}{c} \text{Cell number},\text{fold change}=\mathrm{pre}\left(\%\mathrm{GFP}/\%\text{mCherry}\right)/\\ \text{post}\left(\%\mathrm{GFP}/\%\text{mCherry}\right) \end{array}$$

### 2.10 Analysis of Reactive Oxygen Species

Analysis of intracellular reactive oxygen species (ROS) generation was performed by flow cytometry. Briefly, C2C12 myoblasts were plated in six‐well plates and incubated for 24 h. Cells were then treated with TM (160 ng/mL) or hydrogen peroxide (H_2_O_2_, 1000 mM; Fujifilm Wako Pure Chemical Corporation, Osaka, Japan; Cat# 086‐07445) and N‐acetyl‐L‐cysteine (NAC, 1 mM; Fujifilm Wako Pure Chemical Corporation, Osaka, Japan; Cat# 017‐05131) or dimethyl sulfoxide (DMSO; Fujifilm Wako Pure Chemical Corporation, Osaka, Japan; Cat# 043‐07211) alone for 1 h. Cells were harvested and stained with 500 nM CellROX Green Reagent (Thermo Fisher Scientific, Waltham, MA, USA; Cat# C104492) for 45 min at 37°C and analysed by flow cytometry. The fluorescence signal proportional to cellular ROS levels was excited by a laser at 488 nm and quantified on SH800S cell sorter (Sony Imaging Products & Solutions Inc., Tokyo, Japan).

### 2.11 Haematoxylin and Eosin (H&E) Staining

Immediately after sacrificing mice, TA muscle samples were fixed with 4% paraformaldehyde phosphate buffer solution and replaced with PBS the next day. Tissue section preparation and haematoxylin and eosin (H&E) staining were outsourced to Applied Medical Research (Osaka, Japan). A fluorescence microscope was used for imaging. Cross‐sectional areas of myotubes were automatically quantified using the analysis software supplied by Keyence Corporation, Osaka, Japan. Four randomly selected fields per sample were analysed.

### 2.12 Gene Knockdown by siRNA

The role of Naa35 in C2C12 myoblasts and differentiated myotubes was examined by suppressing Naa35 expression using small interfering RNA (siRNA). A Silencer Select siRNA targeting mouse Naa35 (Thermo Fisher Scientific, Waltham, MA, USA; siRNA ID: s95637) was used for knockdown. For cathepsin K (CTSK) knockdown, a Silencer Select siRNA targeting mouse CTSK (Thermo Fisher Scientific, Waltham, MA, USA; siRNA ID: s64615) was used under the same transfection conditions. C2C12 myoblasts at 70% confluence were plated in six‐well plates 24 h before transfection. The siRNA–liposomal complexes were prepared using Lipofectamine RNAiMAX Reagent (Thermo Fisher Scientific, Waltham, MA, USA; Cat# 13778150) according to the manufacturer's instructions. The siRNA–liposomal complexes were added to C2C12 myoblasts or myotube and incubated for 24 h. Medium was changed after 24 h. Cells were harvested 48 h after transfection. A Silencer Select Negative Control siRNA (Thermo Fisher Scientific, Waltham, MA, USA; Cat# 4390843) was used as a control.

### 2.13 Real‐Time PCR

Total RNA was isolated from C2C12 myoblasts, differentiated myotubes or mouse TA muscles using TRIzol Reagent (Life Technologies, Carlsbad, CA, USA) or the Direct‐zol RNA Miniprep Kit (Zymo Research Corporation, Irvine, CA, USA), according to the manufacturers' instructions. Complementary DNA (cDNA) was synthesized using PrimeScript RT Master Mix (Takara Bio, Shiga, Japan). Quantitative PCR was performed using KAPA SYBR FAST qPCR Master Mix (Kapa Biosystems, Wilmington, MA, USA). Gene expression levels were quantified using the ΔΔCt method and normalized to the housekeeping gene GAPDH. Primer sequences and gene information are listed in Table S1.

### 2.14 Immunoblot

The total protein concentration of the cell or skeletal muscle lysate was determined using the Lowry assay kit (Bio‐Rad Laboratories, Hercules, CA, USA; Cat# 5000112JA). Equal amounts of protein were loaded onto a sulfate‐polyacrylamide gel and separated by electrophoresis. They were transferred to polyvinylidene fluoride (PVDF) membranes (Millipore, Burlington, MA, USA; Cat# IPVH00010). Subsequently, the following primary antibodies were used: anti‐Naa35 (Sigma‐Aldrich, St. Louis, MO, USA; Cat# HPA021547; 1:2000), anti‐CHOP (Proteintech, Rosemont, IL, USA; Cat# 15204‐1‐AP; 1:1000), anti‐phospho‐S6 (Cell Signaling Technology, Danvers, MA, USA; Cat# 4856S; 1:1000), anti‐S6 (Cell Signaling Technology; Cat# 2217; 1:1000), anti‐phospho‐Akt (Cell Signaling Technology; Cat# 9271S; 1:1000), anti‐Akt (Cell Signaling Technology; Cat# 9292S; 1:1000), anti‐ATF6 (Cell Signaling Technology; Cat# 65880T; 1:1,000), anti‐CTSK (Santa Cruz Biotechnology, Dallas, TX, USA; Cat# sc‐48353; 1:1000), anti‐IRS1 (Santa Cruz Biotechnology; Cat# sc‐559; 1:500), anti‐puromycin (EMD Millipore, Burlington, MA, USA; Cat# MABE343; 1:20000), anti‐β‐actin (Sigma‐Aldrich; Cat# A2228; 1:2000) and anti‐GAPDH (EMD Millipore; Cat# MAB374; 1:300). HRP‐conjugated secondary antibodies were used as follows: anti‐rabbit IgG HRP‐linked antibody (Cell Signaling Technology; Cat# 7074S; 1:2000) and anti‐mouse IgG HRP‐linked antibody (Cell Signaling Technology; Cat# 7076S; 1:2000). Immunoreactive bands were detected using Clarity Western ECL substrate (Bio‐Rad Laboratories, Hercules, CA, USA; Cat# 1705060) or Clarity Max Western ECL substrate (Bio‐Rad Laboratories; Cat# 1705062). Densitometric analysis was performed using ImageJ software (National Institutes of Health, Bethesda, MD, USA).

### 2.15 SUnSET Assay

Puromycin dihydrochloride (Sigma‐Aldrich, St. Louis, MO, USA; Cat# P8833) was added to the cell treatment media (1 μM final concentration) for 30 min prior to cell lysis with a standard RIPA buffer. Twenty micrograms of protein was separated in a 10% polyacrylamide gel until the dye front was ∼2 cm from the bottom of the gel. Proteins were transferred to PVDF membranes and subsequently incubated in TBST buffer containing 5% skim milk powder and incubated overnight with a monoclonal puromycin antibody. Membranes were then incubated for 1 h in TBST containing 5% skim milk plus a secondary mouse antibody.

### 2.16 Protein Degradation Assay

Protein degradation was measured by growing siRNA‐transfected C2C12 myoblasts to confluence, switching them to Dulbecco's phosphate‐buffered saline (DPBS; Sigma‐Aldrich, St. Louis, MO, USA; Cat# D8537), and measuring the tyrosine released into the DPBS over time. Protein extracts confirmed there was no significant difference in starting total protein levels between controls and Naa35 siRNA prior to switching to DPBS. The DPBS solutions at the noted timepoints were removed, and then 0.25 V of Trichloroacetic acid (Fujifilm Wako Pure Chemical Corporation, Osaka, Japan; Cat# 203‐04952) was added. The samples were then centrifuged at 4°C and 12 000 × *g* to precipitate and remove proteins. Supernatants (40 μL) were transferred to a 96‐well plate together with 40 μL of tyrosine standards (Fujifilm Wako Pure Chemical Corporation, Osaka, Japan). Sodium carbonate solution (100 μL; 500 mM; Fujifilm Wako Pure Chemical Corporation, Osaka, Japan; Cat# 195‐04555) was added to each well, followed by 20 μL of 0.5 N Folin–Ciocalteu phenol reagent (Sigma‐Aldrich, St. Louis, MO, USA; Cat# F9252) diluted in distilled water. Plates were incubated for 30 min at 37°C, and absorbance was measured at 660 nm. Free tyrosine concentrations were calculated using the standard curve prepared with tyrosine standards.

### 2.17 Grip‐Strength, Inverted Screen Test

Two experiments were conducted to measure exercise strength and muscle strength in mice. First, whole‐limb grip strength was assessed using a grip‐strength meter (NOVERTEC, Tokyo, Japan; MK‐380V). Mice were allowed to grasp a wire mesh with all four limbs, and the tail was gently pulled backward in a horizontal plane until the mouse released its grip. The peak tension (in Newton) recorded at the moment of release was defined as the grip strength. For each mouse, the measurement was repeated three times with at least 30 s of rest between trials, and the mean of the three measurements was used for analysis. This method provides a non‐invasive estimation of overall limb muscle function and has been widely used in rodent studies. Next, after a sufficient interval, the mouse was placed in the center of a stainless‐steel wire mesh screen, the stop clock was started, and the wire mesh was slowly inverted over a period of 2 s. The screen was placed statically at a height of 40–50 cm from a soft padded surface and the time taken for the mouse to fall off the wire mesh was measured.

### 2.18 Statistical Analysis

All data are presented as mean ± SD. The *p* values were calculated by two‐sided unpaired *t*‐tests and one‐way ANOVA with Tukey's multiple comparison test using GraphPad Prism software Version 9. No statistical methods were used to predetermine sample size. Sample size was based on experimental feasibility and sample availability. Samples were processed in random order.

## 3 Results

### 3.1 Caner Cachexia Induces ER Stress in Skeletal Muscles

**FIGURE 1:**
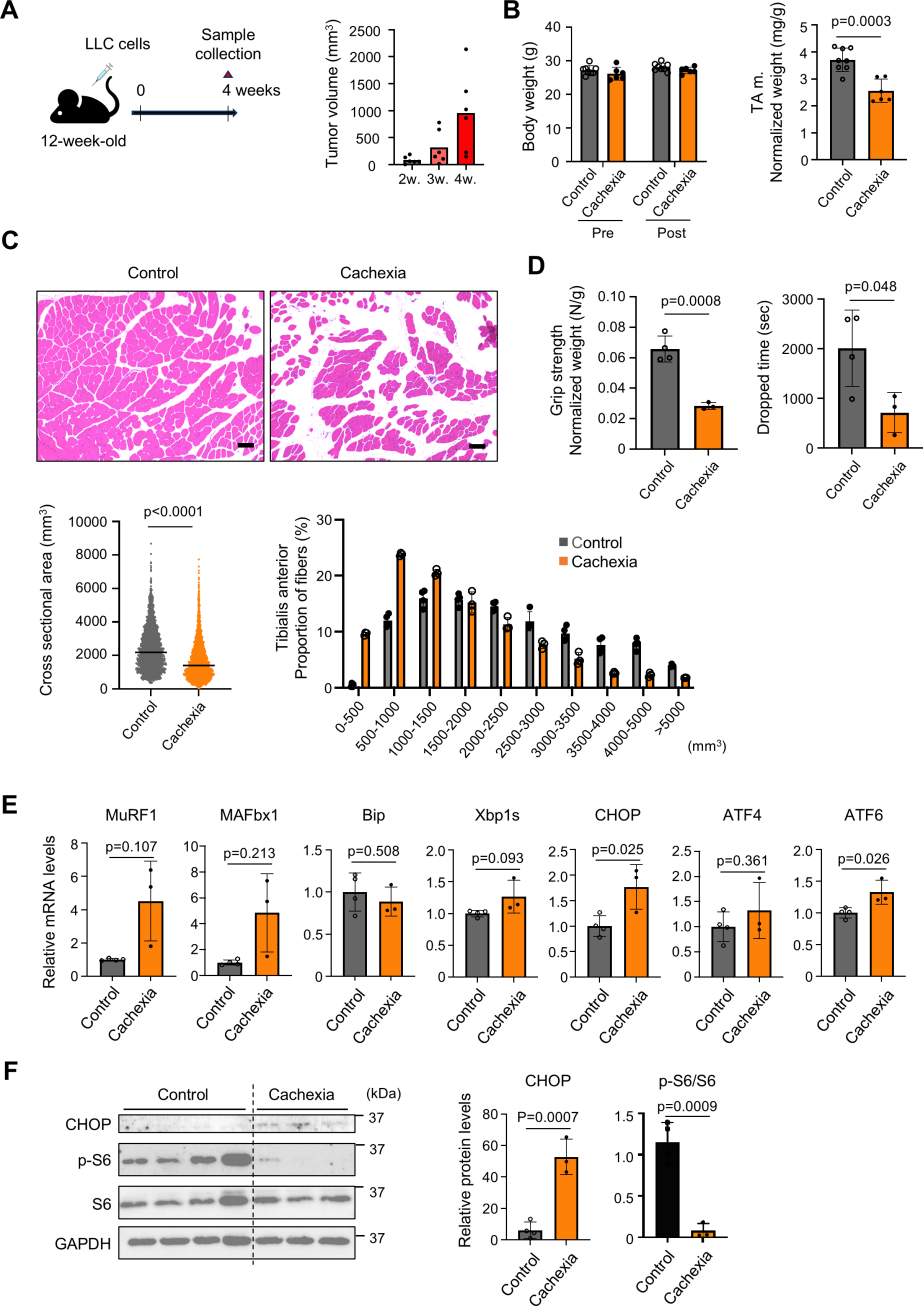
Cancer cachexia induces ER stress in skeletal muscles. (A) Schematic of a mouse model of cancer cachexia with dorsal subcutaneous injection of Lewis lung adenocarcinoma cells (LLC; 1 × 10^5^) in 12‐week‐old mice (left) and tumour size at the indicated time points (right). (B) Changes in body weight and wet weight of TA muscle 4 weeks after LLC injection (control, *n* = 8; cachexia, *n* = 6). Data are expressed as mean ± SD. The *p* values were determined by unpaired *t*‐test. (C) Representative image of TA muscle stained with H&E (top). Scale bar, 100 μm. Cross‐sectional area of TA muscle fibre and its relative frequency distribution (percentage to total fibre number, bottom) (control, *n* = 4; cachexia, *n* = 3). Data are expressed as mean ± SD. (D) Grip strength of limbs measured with a grip strength meter and wire mesh grasping time (control, *n* = 4; cachexia, *n* = 3). Data are expressed as mean ± SD. The *p* values were determined by unpaired *t*‐test. (E) Gene expressions of atrophy‐related genes (MuRF1, MAFbx1) and ER stress markers (Bip, Xbp1s, CHOP, ATF4, ATF6) (control, *n* = 4; cachexia, *n* = 3). Gene expression levels were quantified using the ΔΔCt method and normalized to GAPDH. Relative expressions are shown as fold change compared with the control group. Data are expressed as mean ± SD. The *p* values were determined by unpaired *t*‐test. (F) Immunoblotting images and quantitative data for CHOP, p‐S6, S6 and GAPDH (control, *n* = 4; cachexia, *n* = 3). Data are expressed as mean ± SD. The *p* values were determined by unpaired *t*‐test. The difference in sample numbers is due to mortality in the cachexia model and limited TA muscle tissue availability. See methods for details.

The eukaryotic ER is the site of protein synthesis and folding, lipid synthesis, compound detoxification, glucose metabolism and calcium storage. Of particular importance is the function of proper folding of proteins into three‐dimensional structures; the ER has a quality control mechanism to export only properly folded proteins, and misfolded proteins are degraded by ER‐associated degradation (ERAD). However, the accumulation of unprocessed misfolded proteins disrupts ER homeostasis and threatens cell survival. To cope with this, the cell initiates a signalling cascade called the UPR, which contains three major arms regulated by PERK, IRE1α and ATF6. We first investigated the link between cachexia and ER stress. Mouse models of LLC xenografts show muscle atrophy and are often used as models of cachexia [15]. In this study, LLCs were implanted in the back of 12‐week‐old C57BL/6 male mice, and skeletal muscle was analysed 4 weeks later. This model exhibited tumour growth in a time‐dependent manner (Figure 1A). Although there was no body weight loss at 4 weeks after transplantation, a decrease in wet weight of the TA muscle was observed, accompanied by fatty degeneration of the muscle tissue and a significant reduction in muscle cross‐sectional area, as evidenced by HE staining (Figure 1B,C). Consistent with these morphological changes, functional impairments were observed in the grip‐strength test and inverted screen test in the cachexia mice (Figure 1D). This cachexia model demonstrated the transcriptional upregulation of molecules downstream of ER stress, as well as muscle atrophy‐related E3 ubiquitin ligases (Figure 1E). In addition, the mTOR pathway, which is closely associated with cachexia and ER stress [16], was markedly suppressed in the TA muscle of LLC‐bearing mice (Figure 1F).

### 3.2 Prolonged ER Stress Recapitulates Atrophy in Myotube

**FIGURE 2:**
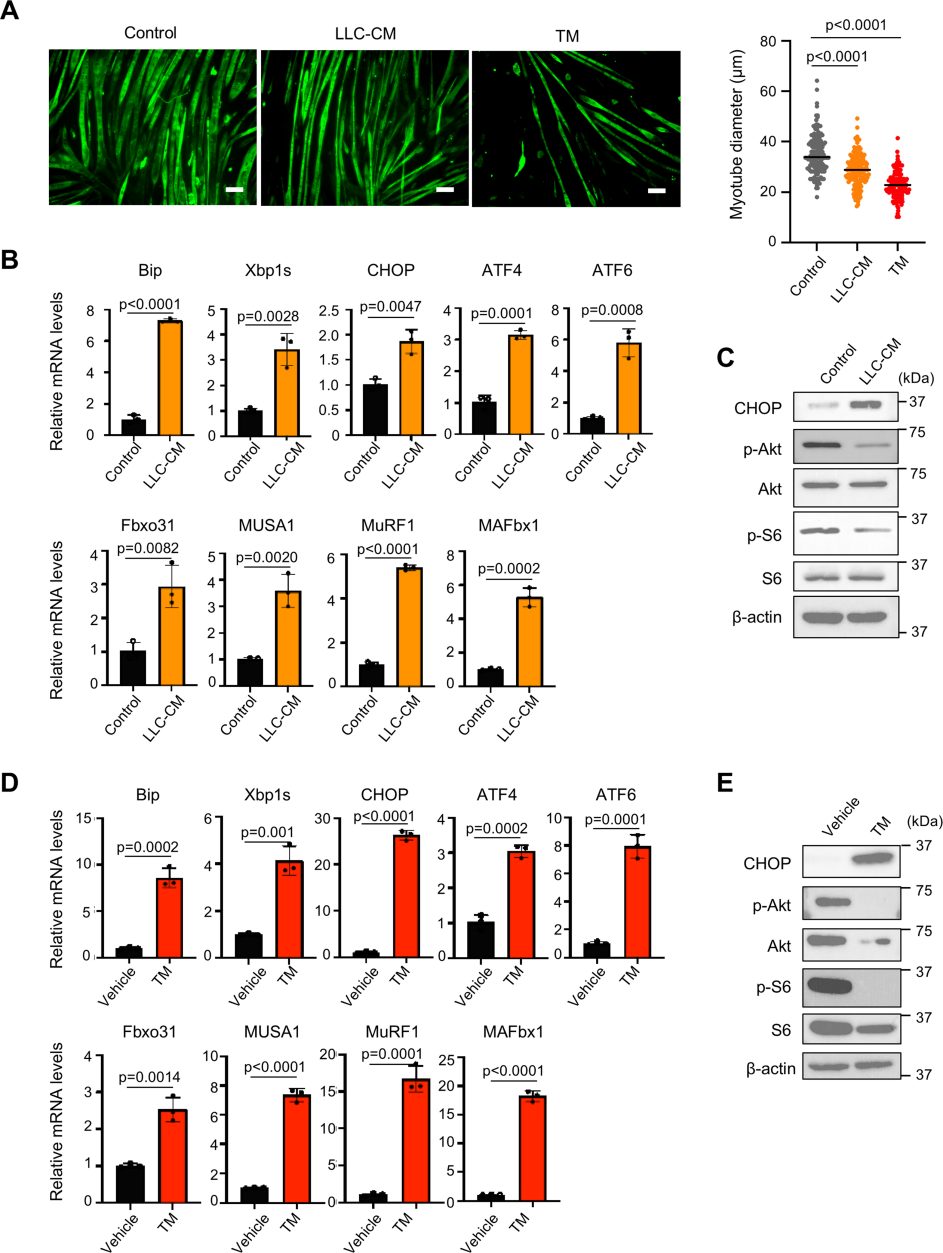
Prolonged ER stress recapitulates atrophy in myotubes. (A) Immunofluorescence staining for desmin and quantification of diameter in differentiated myotubes treated with tunicamycin (TM) or 50% LLC conditioned medium (LLC‐CM) for 72 h. Scale bar, 100 μm. Data are expressed as median. The *p* values were determined by one‐way ANOVA with Tukey's multiple comparison test. (B) Gene expressions of ER stress markers (Bip, Xbp1s, CHOP, ATF4, ATF6) and atrophy‐related genes (Fbxo31, MUSA1, MuRF1, MAFbx1) in myotubes after 50% LLC‐CM treatment for 72 h. Gene expression levels were quantified using the ΔΔCt method and normalized to GAPDH. Relative expressions are shown as fold change compared with the control group. Data are expressed as mean ± SD. The *p* values were determined by unpaired *t*‐test. (C) Immunoblot of myotubes after treatment with 50% LLC‐CM for 72 h. (D) Gene expressions of ER stress markers and atrophy‐related genes in myotubes after TM treatment for 72 h. Gene expression levels were quantified using the ΔΔCt method and normalized to GAPDH. Relative expressions are shown as fold change compared with the vehicle group. Data are expressed as mean ± SD. The *p* values were determined by unpaired *t*‐test. (E) Immunoblot of myotubes after TM treatment for 72 h.

In vitro, cachexia was reproduced in C2C12 differentiated myotubes. The atrophic alteration was observed in myotubes when treated with LLC‐CM as well as tunicamycin, an ER stress inducer (Figure 2A). In myotubes treated with LLC‐CM, the transcription of UPR signals and muscle atrophy–related genes was upregulated (Figure 2B). As observed in the in vivo model, the activities of mTOR and its upstream regulator, Akt, were found to be diminished in response to treatment with LLC‐CM (Figure 2C). Prolonged tunicamycin treatment, which robustly activated UPR signals, also induced atrophy‐related gene expression and Akt/mTOR pathway inhibition (Figure 2D,E). These findings suggest that LLC‐mediated cachexia is reproduced in myotubes and that ER stress contributes to this process.

### 3.3 Genome‐Wide CRISPR Screen Identifies NatC as an ER Stress–Resistant Regulator

**FIGURE 3:**
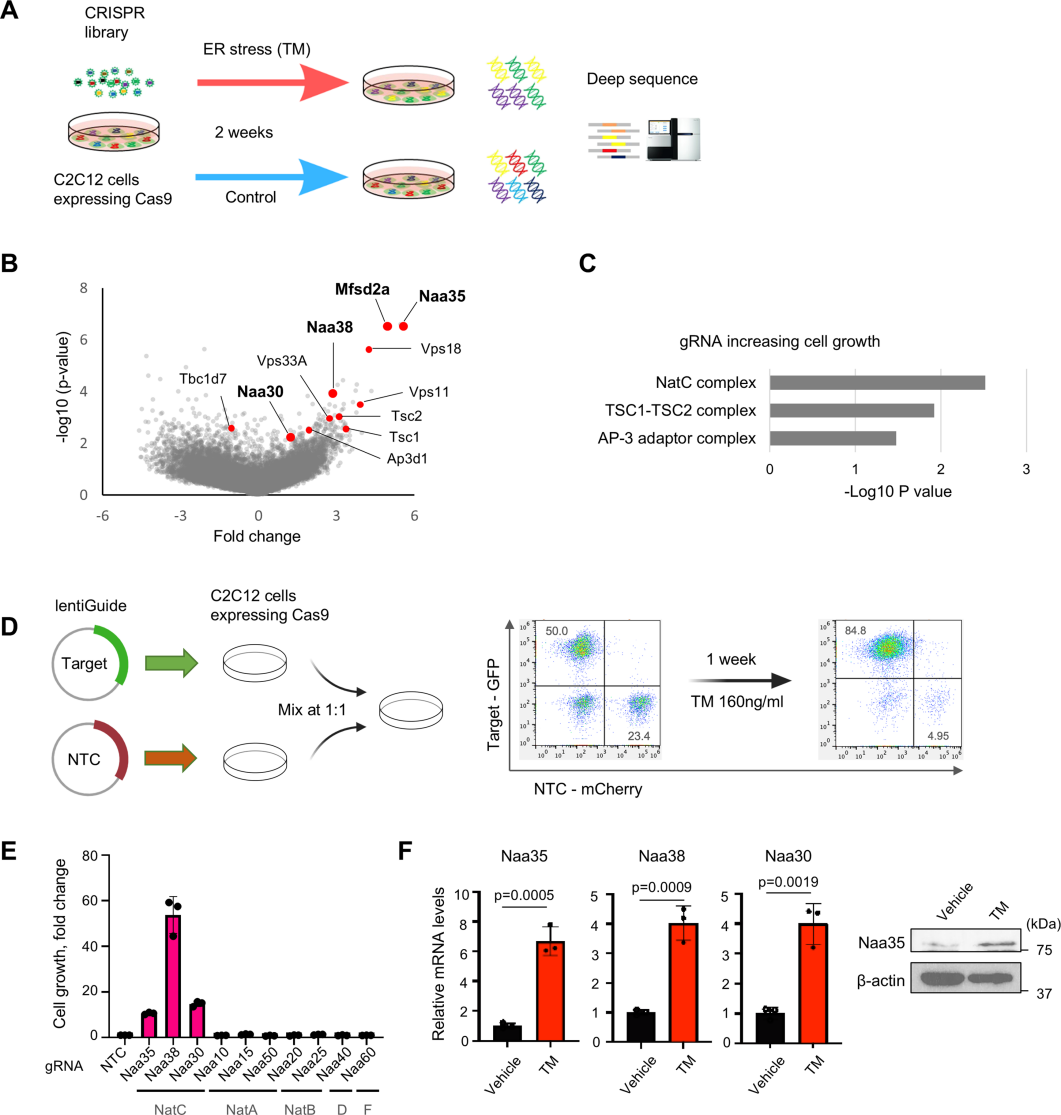
Genome‐wide CRISPR screen identifies NatC as an ER stress–resistant regulator. (A) Schematic of a CRISPR library screen to identify genes that modulate cell growth inhibition under ER stress. The library was transduced by lentivirus into mouse myoblast C2C12 myoblasts, and the cells were treated with tunicamycin (160 ng/mL) for 2 weeks. The cells were harvested, and the extracted gRNAs underwent deep sequencing. (B) Volcano plot analysis of the CRISPR screen showing gRNAs that affected ER stress–resistant cell growth. (C) GSEA of modulators for cell growth under ER stress. The top 1% of genes in RRA score were analysed. Representative functional categories and Bonferroni‐corrected *p* values are shown. (D) Schematic of assay evaluating the effect of individual gRNA. The gRNA against candidate genes and NTC was labelled with GFP and mCherry, respectively. These gRNAs‐infected cells were cultured in mixed condition with treatment of 160 ng/mL tunicamycin (TM) for 7 days, and GFP and mCherry‐positive populations were analysed by flow cytometry. (E) The effect of Nat‐related genes on cell growth under TM treatment as compared with NTC. Data are expressed as mean ± SD. The *p* values were determined by unpaired *t*‐test. (F) The RNA and protein expression of NatC components in C2C12 myoblasts treated with TM for 72 h. Gene expression levels were quantified using the ΔΔCt method and normalized to GAPDH. Relative expressions are shown as fold change compared with the vehicle group.

In order to understand the intricate mechanisms underlying ER stress–mediated atrophy, we conducted a genome‐wide CRISPR library screening in the context of cell growth inhibition under prolonged ER stress. This approach allowed us to perform pooled library screening. The library was transduced by lentivirus into mouse C2C12 myoblasts, and the cells were treated with tunicamycin as ER stress for 2 weeks. Cells from the treatment group and the control group were collected, and the extracted genomic DNA underwent gRNAs deep sequence and analysis (Figure 3A). We systematically analysed genes whose gRNAs affected ER stress–resistant cell growth using the MAGeCK algorithm (Figure 3B). One of the top‐hit genes was Mfsd2a, a putative plasma membrane transporter for tunicamycin [17], supporting the robustness of the screen. Unbiased gene set enrichment analysis (GSEA) of gRNAs that significantly modulated the sensitivity to tunicamycin identified NatC as the most important target class (Figure 3C). In eukaryotes, five conserved NATs (NatA to NatE) have been identified that catalyse the acetylation of the N‐terminus of substrate proteins, thereby affecting protein homeostasis [18]. The effect of individual gRNA was analysed by concurrent culture with control cells. Cells with target gRNA and NTC gRNA were labelled with GFP and mCherry, respectively, and cultured together under tunicamycin treatment for 7 days. The change in each population ratio was quantified using flow cytometry (Figure 3D). In this assay, it was observed that cell growth was preserved when gRNA targeting NatC components, specifically Naa30, Naa35 and Naa38, was administered. In contrast, gRNA targeting other Nat family members, including NatA, NatB, NatD and NatF, did not significantly affect sensitivity to tunicamycin (Figure 3E). The gene expression of NatC components and the protein level of Naa35 were upregulated under tunicamycin treatment (Figure 3F), collectively indicating that NatC was upregulated by tunicamycin and involved in the impairment of cell growth. Furthermore, the restoration of cell growth was observed in thapsigargin‐induced ER stress (Figure S1B). The protective effect of NatC deletion was not specific to tunicamycin but rather extended to other forms of ER stress. In contrast, NatC deletion‐mediated restoration of cell growth under treatment with tunicamycin was not replicated in Neuro2A neuroblasts (Figure S1C). The protective effect of NatC deletion was not universally observed in other cell types.

ER stress is intimately linked with oxidative stress through its interaction with mitochondria. Consistently, tunicamycin treatment resulted in elevated ROS production in our experimental setting (Figure S2A). Accordingly, an investigation was conducted to ascertain the protective effect under other stress conditions, including treatments with hydrogen peroxide or doxorubicin. The deletion of each component of NatC had no effect on oxidative or genotoxic stress, indicating that NatC involvement is specific to ER stress (Figure S2B).

### 3.4 NatC Silencing Mitigates ER Stress–Related Myotube Atrophy

**FIGURE 4:**
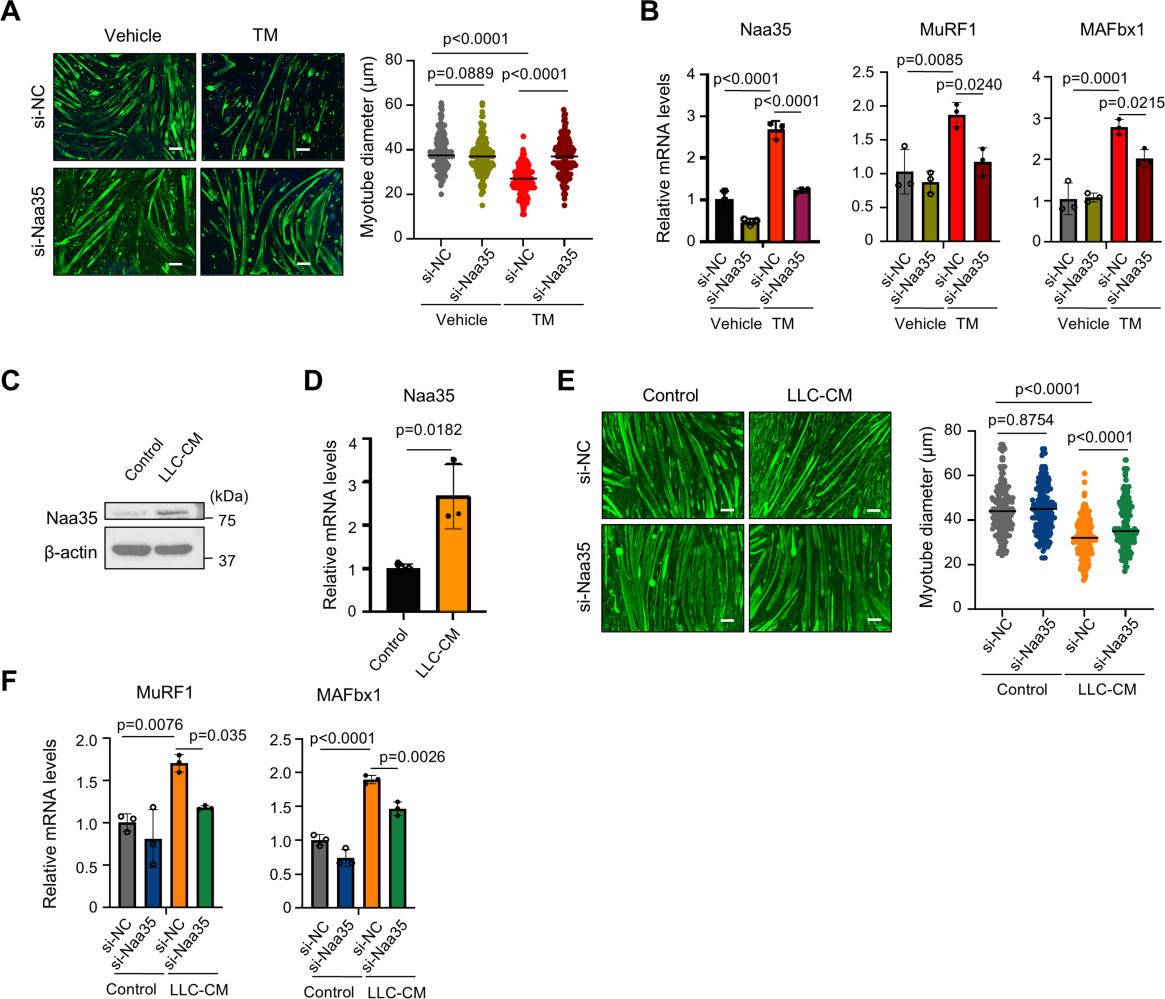
NatC silencing mitigates myotube atrophy associated with ER stress. (A) Immunostaining images of tunicamycin (TM)‐stimulated Naa35‐silenced or control myotubes and these diameters. (TM, 160 ng/mL, 48 h). Scale bar, 100 μm. Data are expressed as median. The *p* values were determined by one‐way ANOVA with Tukey's multiple comparison test. (B) qPCR analysis of Naa35 and atrogenes (MuRF1 and MAFbx1). Gene expression levels were quantified using the ΔΔCt method and normalized to GAPDH. Relative expression is shown as fold change compared with the vehicle‐treated si‐NC group. Data are expressed as mean ± SD. The *p* values were determined by one‐way ANOVA with Tukey's multiple comparison test. (C,D) Immunoblot analysis of Naa35 and qPCR analysis of Naa35 in whole cell lysates of 50% LLC‐CM‐stimulated myotube for 72 h. Gene expression levels were quantified using the ΔΔCt method and normalized to GAPDH. Relative expressions are shown as fold change compared with the control group. Data are expressed as mean ± SD. The *p* values were determined by unpaired *t*‐test. (E) Immunostaining images of myotubes and diameter after 50% LLC‐CM treatment for 72 h. Scale bar, 100 μm. Data are expressed as median. The *p* values were determined by one‐way ANOVA with Tukey's multiple comparison test. (F) qPCR analysis of atrogenes (MuRF1 and MAFbx1). Gene expression levels were quantified using the ΔΔCt method and normalized to GAPDH. Relative expressions are shown as fold change compared with the control si‐NC group. Data are expressed as mean ± SD. The *p* values were determined by one‐way ANOVA with Tukey's multiple comparison test.

We next investigate whether the protective impact of NatC deletion on ER stress is reproduced in the context of differentiated myotube atrophy. Differentiated myotubes were transfected with siRNA and 2 days later treated with tunicamycin for 2 days. The knockdown of Naa35 was observed to morphologically alleviate the tunicamycin‐induced atrophy of myotubes, though no change was noted in the control myotubes treated with PBS (Figure 4A). Consistently, the upregulation of atrophy‐related MuRF1 and MAFbx1 transcription was also mitigated (Figure 4B). In the LLC‐CM‐mediated myotube atrophy model, the mRNA and protein levels of Naa35 were upregulated (Figure 4C,D), similar to the tunicamycin treatment (Figure 3F,G). The knockdown of Naa35 resulted in the preservation of myotube diameter size and a reduction in the expression of MuRF1 and MAFbx1 (Figure 4E,F).

We additionally examined the ER stress and Naa35‐mediated myotube atrophy using a distinct in vitro cachexia model with C26 colon carcinoma cells. The conditioned medium of C26 colon carcinoma cells (C26‐CM) induced myotube atrophy (Figure S3A) with the upregulation of UPR signal and muscle atrophy–related gene transcription (Figure S3B). This process was accompanied by the downregulation of the mTOR/Akt pathway (Figure S3C). As was the case with LLC‐CM, C26‐CM also activated the Naa35 expression (Figure S3B,C). In addition, the knockdown of Naa35 led to the restoration of myotube size and the prevention of MuRF1 and MAFbx1 upregulation (Figure S3D,E). These results indicate that in vitro myotube atrophy via ER stress and Naa35 is not specific to LLC and may occur more generally.

The impact of NatC was also assessed in a steroid‐induced atrophy model. The administration of dexamethasone to myotubes did not result in the elevated expression of ER stress–related genes or NatC components (Figure S4A). In this model, the knockdown of Naa35 did not result in any changes in the atrophy of myotubes (Figure S4B), suggesting that Naa35 contributed to ER stress–related myotube atrophy.

### 3.5 ATF6 Upregulates NatC Expression Under ER Stress

**FIGURE 5:**
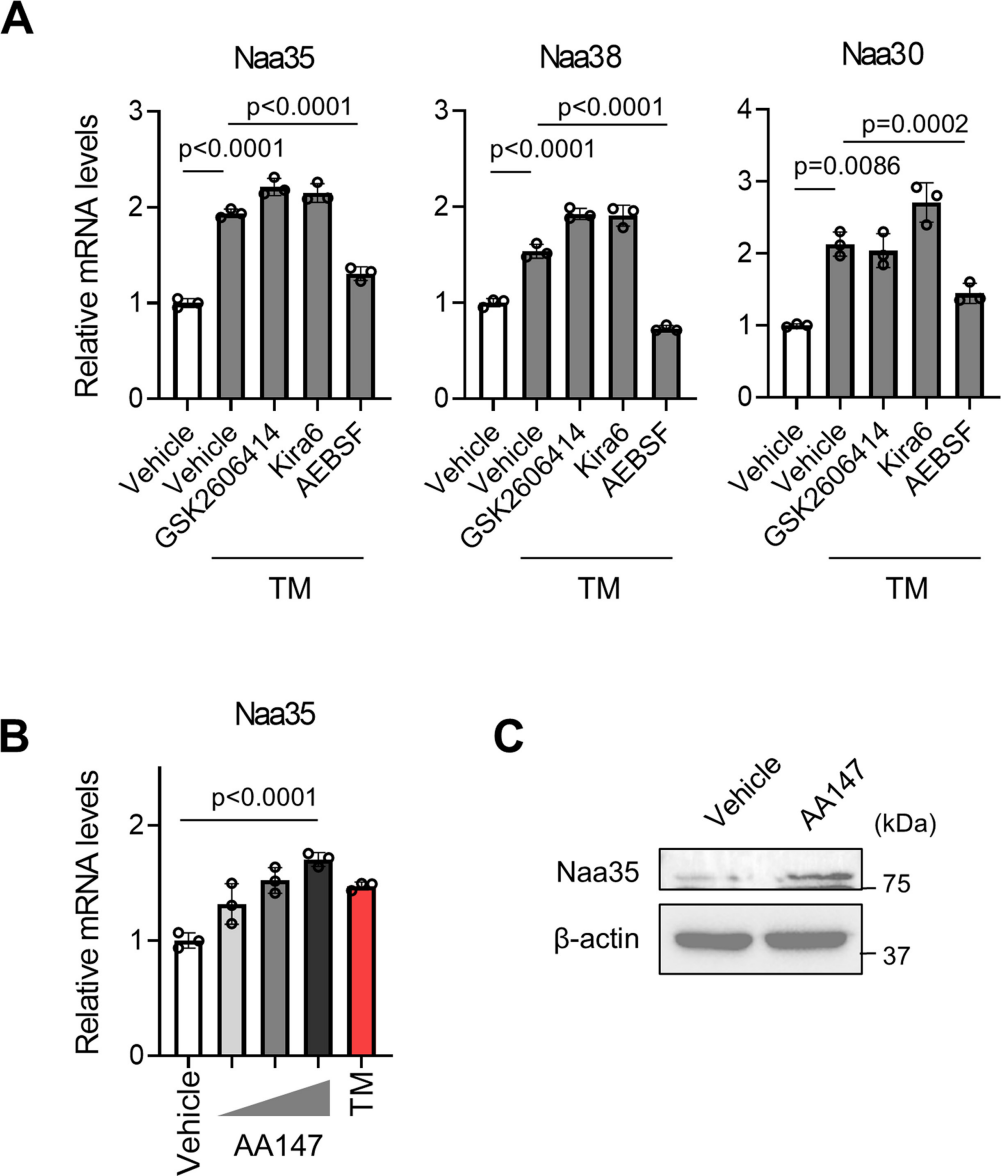
ATF6 upregulates NatC expression under ER stress. (A) Gene expressions of NatC components, Naa35, Naa38 and Naa30 in C2C12 myoblasts treated with tunicamycin (TM; 160 ng/mL) and inhibitors, GSK2606414 (1 μM), Kira6 (3 μM) and AEBSF (400 μM) for 15 h. Gene expression levels were quantified using the ΔΔCt method and normalized to GAPDH. Relative expressions are shown as fold change compared with the vehicle group. Data are expressed as mean ± SD. The *p* values were determined by one‐way ANOVA with Tukey's multiple comparison test. (B) Gene expression of Naa35 after treatment with ATF6 activator, AA147 (up to 5 μM) or TM (160 ng/mL) for 15 h. Gene expression levels were quantified using the ΔΔCt method and normalized to GAPDH. Relative expressions are shown as fold change compared with the vehicle group. Data are expressed as mean ± SD. The *p* values were determined by one‐way ANOVA with Tukey's multiple comparison test. (C) Immunoblot analysis of ATF6, Naa35 and β‐actin after treatment of AA147 (5 μM) or DMSO.

The transcription of NatC components was upregulated by tunicamycin treatment (Figure 3F,G). There are three principal ER stress sensors—PERK, ATF6 and IRE1α—which detect misfolded proteins in the ER lumen and trigger a coordinated transcriptional response to mitigate the stress. C2C12 myoblasts were treated with tunicamycin together with each sensor inhibitor, GSK for PERK [19], Kira6 for IRE1α [20] and AEBSF for ATF6 [21]. Among these inhibitors, AEBSF abolished the tunicamycin‐mediated upregulation of Naa35, Naa38 and Naa30 (Figure 5A). Furthermore, the attenuation of NatC transcription mediated by AEBSF was also observed in thapsigargin‐induced ER stress (Figure S5). These results indicate that the ATF6 pathway is involved in NatC transcription in response to ER stress. Subsequently, cells were treated with AA147, a compound that activates the ATF6 pathway [22]. AA147 increased Naa35 transcription in a dose‐dependent manner, and Naa35 protein was also upregulated, providing further evidence that ATF6 regulated the transcription of NatC (Figure 5B,C).

### 3.6 NatC Stabilizes Cathepsin K and Induces Cachexia Through IRS1‐Akt/mTOR Pathway

To understand the detailed molecular mechanism underlying NatC‐mediated muscle atrophy, we first focused on the potential impact on the three principal ER stress sensor pathways. PERK upregulates CHOP transcription via ATF4. IRE1α modulates splicing of XBP1 mRNA, and the resultant active form of XBP1 functions as a promoter. ATF6 is proteolytically cleaved to become active, which then promotes the expression of target genes, including HSPA5 [23]. Deletion of Naa35 did not result in a reduction in the expression of CHOP, HSPA5 and spliced Xbp1 upon treatments with tunicamycin and thapsigargin (Figure S6A), indicating that NatC affected pathways other than ER stress sensors.

**FIGURE 6:**
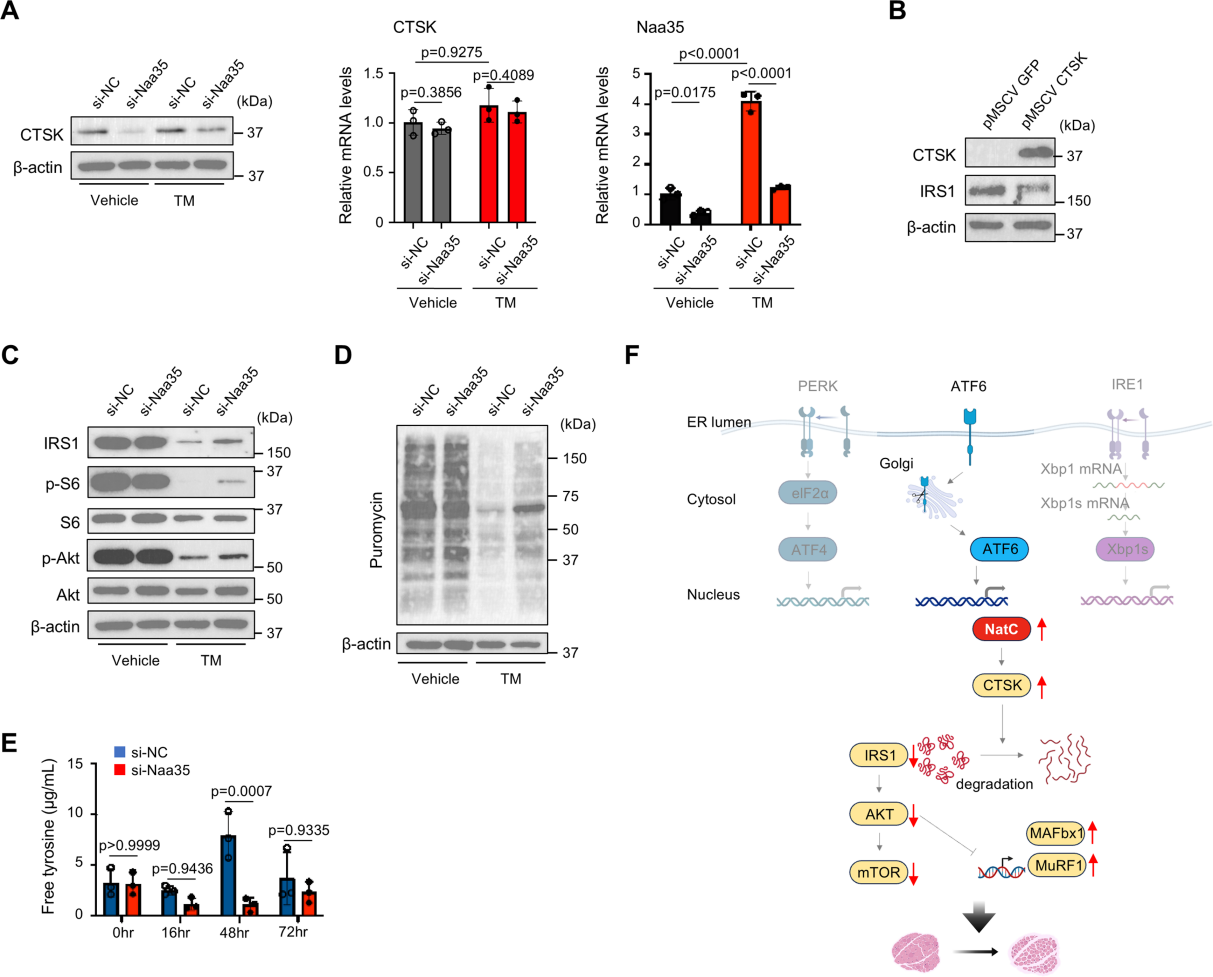
NatC contributes to cathepsin K stabilization and atrophy through IRS1‐Akt/mTOR pathway. (A) C2C12 myoblasts were transfected with siRNA targeting Naa35 or negative control, and 2 days later treated with tunicamycin (TM; 160 ng/mL) for 72 h. Immunoblot and qPCR analysis of CTSK and Naa35 in whole‐cell lysates. Gene expression levels were quantified using the ΔΔCt method and normalized to GAPDH. Relative expression is shown as fold change compared with the vehicle‐treated group. Data are expressed as mean ± SD. The *p* values were determined by one‐way ANOVA with Tukey's multiple comparison test. (B) Immunoblot analysis of CTSK, IRS1 and β‐actin in whole‐cell lysates of C2C12 myoblasts overexpressing CTSK with retrovirus. (C) Immunoblot analysis of IRS1, p‐S6, S6, p‐Akt, Akt and β‐actin in whole‐cell lysates of C2C12 myoblasts treated with siRNA targeting Naa35 and TM in the same protocol as (A). (D) The SUnSET assay in cultured myotubes. Myotubes were transfected with siRNA targeting Naa35 or negative control and followed by the treatment with vehicle alone or tunicamycin (160 ng/mL) for 72 h. After pre‐treatment with 100 μM of puromycin for 30 min, immunoblots were performed to evaluate the levels of puromycin‐tagged proteins. (E) Protein degradation rates measured by the release of free tyrosine in indicated time points in C2C12 myoblasts treated with siRNA targeting Naa35 and tunicamycin in the same protocol as (A). Data are expressed as mean ± SD. The *p* values were determined by one‐way ANOVA with Tukey's multiple comparison test. (F) Role of NatC in ER stress–mediated muscle wasting in cancer cachexia.

The NatC is responsible for the acetylation of approximately 30% of the total proteins, thereby modulating protein turnover and function. This process depends on the N‐terminus of the protein, where Met‐Ile, Met‐Leu, Met‐Trp or Met‐Phe is susceptible to NatC acetylation [24]. The mTOR pathway affects the rate of protein synthesis and cell survival and is involved in the pathogenesis of muscle hypertrophy. The IRS1 protein, which is upstream of the Akt/mTOR pathway, is known to be degraded by CTSK [25], which contains a Met‐Trp at its N‐terminus (Figure S6B). The knockdown of Naa35 in C2C12 myoblasts resulted in a reduction in CTSK protein levels in both the control and tunicamycin treatment groups. In contrast, no significant alteration was observed in the CTSK transcription, indicating the increased degradation of CTSK protein without acetylation by NatC (Figure 6A). The overexpression of CTSK resulted in a decrease in the amount of IRS1 protein (Figure 6B), which is consistent with the previous report that IRS1 is the substrate of CTSK [25]. We further examined the IRS1‐Akt/mTOR pathway. Tunicamycin treatment decreased IRS1 protein expression and consequent Akt and S6 phosphorylation, and these changes were alleviated by the knockdown of NatC (Figure 6C). In conjunction with mTOR activity, the SUNSET assay showed that ER stress–mediated reduction of protein synthesis was restored by Naa35 knockdown (Figure 6D). Furthermore, in the free tyrosine assay, which reflects protein degradation, tunicamycin treatment increased the level of free tyrosine, but knockdown of Naa35 normalized this increase (Figure 6E). An investigation was conducted to determine the role of CTSK downstream of Naa35. The knockdown of CTSK significantly attenuated LLC‐CM‐induced myotube atrophy, as evidenced by the preservation of myotube diameter and the repression of MuRF1 and MAFbx1, as compared to control siRNA‐treated cells (Figure S6C,D). Importantly, CTSK knockdown also maintained the expression of IRS1 and subsequent activation of the Akt/mTOR signalling pathway, as indicated by sustained phosphorylation of Akt and S6 (Figure S6D). These results collectively suggest that NatC silencing reduces CTSK protein expression and consequent IRS1 proteolysis, leading to the activation of Akt and mTOR. Preserved Akt prevented the transcriptional activation of MAFbx1 and MuRF1, and mTOR preserved the balance of protein synthesis and degradation (Figure 6F).

### 3.7 Naa35 Silencing Prevents Skeletal Muscle Atrophy in a Mouse Model of Cancer Cachexia

**FIGURE 7:**
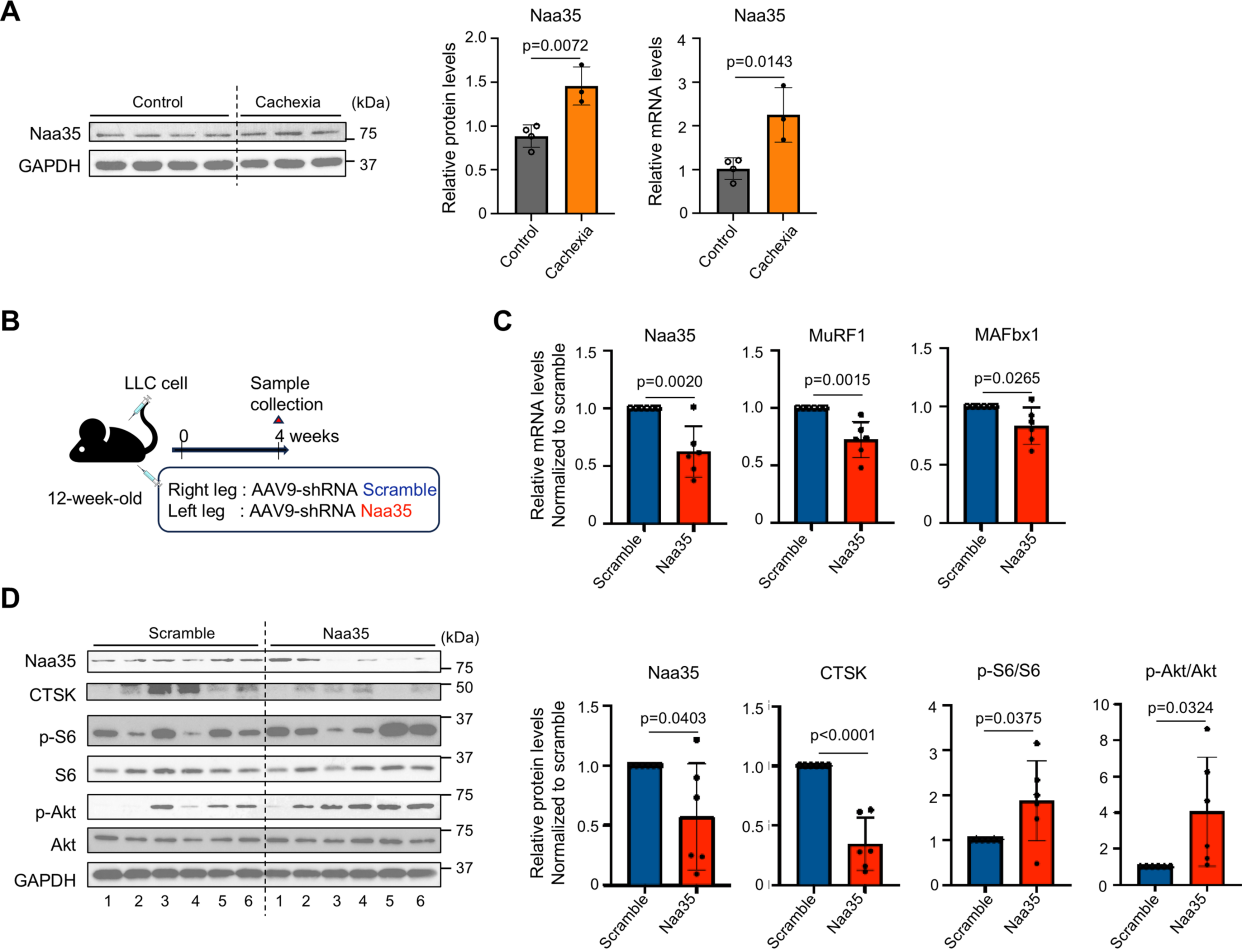
Naa35 silencing preserves the activity of Akt/mTOR pathway in a mouse model of cancer cachexia. (A) Protein and mRNA expression levels of Naa35 in the TA muscle from mice bearing LLC for 4 weeks (control, *n* = 4; cachexia, *n* = 3). Gene expression levels were quantified using the ΔΔCt method and normalized to GAPDH. Relative expressions are shown as fold change compared with the control group. Data are expressed as mean ± SD. The *p* values were determined by unpaired *t*‐test. (B) Concurrently with the transplantation of LLC cells, AAVs carrying shRNA scramble and shRNA Naa35 were injected into the right and left legs, respectively (*n* = 6 per group). (C) Gene expressions of Naa35, MuRF1 and MAFbx1 in the TA muscle. Gene expression levels were quantified using the ΔΔCt method and normalized to GAPDH. Relative expressions are shown as fold change compared with the control group. Data are expressed as mean ± SD. The *p* values were determined by unpaired *t* test. (D) Immunoblotting images and quantitative data of Naa35, CTSK, p‐S6/S6 and p‐Akt/Akt in the TA muscle. Relative expressions are shown as fold change compared with the control group. Data are expressed as mean ± SD. The *p* values were determined by unpaired *t*‐test.

**FIGURE 8:**
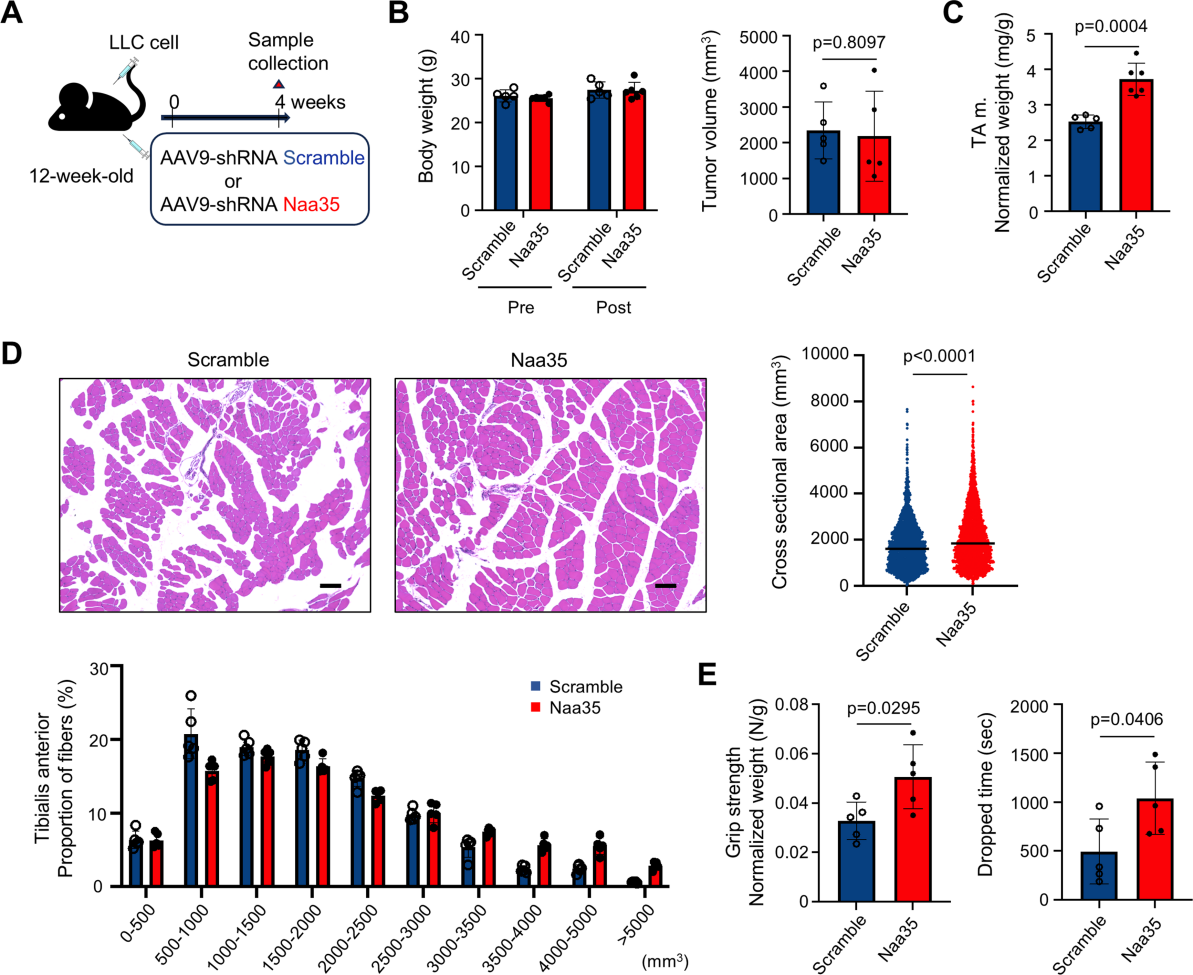
Naa35 silencing prevents skeletal muscle atrophy in a mouse model of cancer cachexia. (A) Simultaneously with the transplantation of LLC cells, mice received intramuscular injections of AAV9‐shRNA scramble or AAV9‐shRNA Naa35 into the TA muscle and were analysed 4 weeks after injection (*n* = 5 per group). (B,C) Whole mouse body weight, tumour size and wet weight of TA muscle. Data are expressed as mean ± SD. The *p* values were determined by unpaired *t*‐test. (D) H&E staining images of TA muscle and cross‐sectional area of muscle fibres. Data are expressed as median (right) or mean ± SD (bottom). The *p* values were determined by unpaired *t*‐test. Scale bar, 100 μm. (E) Grip strength of limbs measured with a grip strength meter and wire mesh grasping time. Data are expressed as mean ± SD. The *p* values were determined by unpaired *t*‐test.

We first confirmed that Naa35 expression at both mRNA and protein levels was increased in the mouse skeletal muscle 4 weeks after LLC transplantation (Figure 7A). Next, in conjunction with LLC transplantation into 3‐month‐old mice, AAV carrying shRNA scramble or Naa35 was also delivered into the right and left anterior tibialis muscles, respectively (Figure 7B). The mRNA levels of Naa35 and atrophy‐related MAFbx1 and MuRF1 were reduced in the Naa35 knockdown (Figure 7C). Furthermore, CTSK expression was diminished, and phosphorylation of Akt and S6 was enhanced in the Naa35‐knockdown muscles (Figure 7D). These results suggest that Naa35 silencing contributes to the reduction of CTSK and the consequent preservation of Akt/mTOR signalling activity in vivo. Subsequently, the protective effect of Naa35 silencing was evaluated by injecting AAV carrying shRNA scramble or Naa35 into both anterior tibialis muscles of 3‐month‐old LLC‐transplanted mice (Figure 8A). At 4 weeks post injection, no differences in body weight and tumour volume were observed between the scramble and Naa35 groups (Figure 8B). In contrast, the wet weight of the TA muscle was maintained in Naa35 knockdown (Figure 8C), with no evident atrophy or fatty degeneration observed in the histological analysis of muscle cross‐sectional area. Quantitative analysis demonstrated that Naa35 knockdown increased myotubular cross‐sectional area (Figure 8D). In addition, physiological muscle strength was assessed through the measurement of grip strength in the limbs and the inverted screen test. Mice injected with shRNA targeting Naa35 showed increased grip strength and longer hanging time on screen (Figure 8E). These results indicate that knockdown of Naa35 prevents both morphological and functional deterioration of skeletal muscles in cancer cachexia.

## 4 Discussion

Cachexia is a multifaceted syndrome characterized by progressive loss of skeletal muscle mass and function, contributing to increased morbidity and mortality in end‐stage diseases such as cancer, chronic obstructive pulmonary disease (COPD), chronic kidney disease and heart failure. In developed countries where epidemiologic data are available, nearly 50%–80% of cancer patients suffer from cachexia, with mortality rates ranging from 20% to 80% [26]. Cancer cachexia accelerates the complications and diminishes the therapeutic efficacy of anti‐cancer treatments. Therefore, elucidating the mechanisms of muscle wasting and facilitating the development of new therapeutic approaches for cancer patients is a crucial research priority. Recent studies have shown that ER stress and the UPR are increased in human skeletal muscle wasting. Muscles from cancer cachexia patients showed increased expression of ER stress markers and all three branches of UPR and fewer ER stress markers; the IRE1‐α arm was also upregulated in COPD cachexia [3]. Accumulation of misfolded proteins and aberrant calcium levels result in ER stress. Adaptive UPR improves protein folding and restores calcium homeostasis, thereby mitigating ER stress and maintaining muscle health. However, chronic activation of UPR can maladaptively induce muscle atrophy via a catabolic imbalance between protein synthesis and degradation and stimulation of apoptosis [27]. The pathological role of UPR in muscle atrophy has been investigated in the mouse model with chemical and genetic interventions. The present study unbiasedly identified NatC as a determinant of muscle atrophy in the context of ER stress in cancer cachexia, where silencing of NatC mitigated the atrophic phenotypes. Conversely, a previous study reported that 4‐PBA, a chemical chaperone known as a pan‐inhibitor of ER stress, further reduced muscle mass in LLC‐bearing mice and also induced rapid atrophy even in naïve mice. Mechanistically, the global inhibition of the UPR reduced the activity of the Akt/mTOR pathway and increased the expression of components of the atrophy‐associated ubiquitin proteasome system (UPS) and autophagy‐lysosome system (ALS) in LLC‐bearing mice [28]. This result contrasts with the protective effect of selective inhibition of the ATF6–NatC axis observed in the present study, suggesting that each branch of the UPR may differentially affect the Akt/mTOR pathway. The activation of ER stress markers and the UPR, including CHOP, was also observed in denervation‐mediated atrophy, in which CHOP prevented the excessive loss of muscle mass via inhibition of autophagy. The underlying mechanism was unknown, but UPS activity, which is mainly regulated by Akt, was unaltered in this context, suggesting that UPR can lead to pathways other than Akt/mTOR signalling [29].

One important unresolved question is what upstream tumour‐derived factors initiate ER stress and activate the NatC‐ATF6 pathway in skeletal muscle. In this study, we showed that conditioned medium derived from LLC cells was sufficient to induce ER stress and reduce myotube diameter. This finding indicates that soluble tumour‐derived factors can directly trigger ER stress–associated catabolic signalling in muscle cells. Consistent with this notion, Bohnert et al. demonstrated that LLC conditioned medium activates UPR pathways in C2C12 myotubes, including upregulation of GRP78/BiP, CHOP and phosphorylation of eIF2α, providing direct evidence that LLC‐secreted factors are sufficient to induce ER stress in muscle cells [28]. Although this study did not identify the precise molecules responsible for ER stress induction, several classes of tumour‐derived mediators have been implicated in previous cancer cachexia studies. These include inflammatory cytokines such as IL‐6 and TNF‐α, which activate ER stress signalling through JAK/STAT3 and IRE1α–XBP1 pathways [30]. In addition, metabolic stress–related mediators, particularly ROS, are known to disrupt protein folding and ER Ca^2+^ homeostasis and thereby activate ER stress and the UPR in muscle cells and other cell types [31, 32]. Furthermore, TGF‐β family ligands such as GDF15 and Activin A, which are elevated in cancer cachexia, contribute to muscle atrophy and stress signalling via Smad2/3‐dependent pathways [33]. Collectively, these findings support a model in which multiple tumour‐derived soluble factors present in LLC conditioned medium converge on ER stress and UPR activation in skeletal muscle, thereby engaging the ATF6–NatC axis and promoting muscle wasting. Identification of the specific upstream mediators responsible for activating this pathway will be an important goal for future studies.

In contrast to cancer cachexia, where ER stress and UPR activation represent major upstream drivers of muscle degeneration, dexamethasone‐induced muscle atrophy is predominantly mediated by ER stress–independent pathways. Glucocorticoids strongly activate FOXO transcription factors, leading to robust induction of the E3 ubiquitin ligases Atrogin‐1/MAFbx and MuRF1, which constitute the core machinery of the ubiquitin–proteasome system (UPS) responsible for accelerated proteolysis in skeletal muscle [34, 35]. Because this UPS‐dominated catabolic program proceeds largely independently of ER stress signalling, Dex‐treated myotubes in our study did not exhibit appreciable activation of ER stress markers or NatC expression. Consequently, Naa35 knockdown failed to ameliorate Dex‐induced atrophy, in contrast to its protective effect in cancer cachexia. These observations highlight a fundamental mechanistic difference between tumour‐associated and steroid‐induced muscle wasting and further support the conclusion that the function of NatC uncovered in this study is specific to ER stress–driven muscle atrophy in cancer cachexia. To further examine whether activation of the ER stress–NatC axis is specific to the LLC model or represents a more general feature of cancer cachexia, we extended our analysis to an independent cachectic tumour cell line, C26 colon carcinoma cells. Using conditioned medium derived from C26 cells, we observed robust induction of myotube atrophy in C2C12 cultures, accompanied by upregulation of ER stress markers, muscle atrophy–related genes and NatC components. These findings demonstrate that tumour‐derived factors from distinct cachectic cancers are capable of activating the ER stress–NatC pathway in skeletal muscle cells. Although in vivo validation using C26 tumour‐bearing mice would be an important future step, the current in vitro data provides strong independent evidence that ER stress–associated NatC activation is not restricted to the LLC model, but rather reflects a shared mechanism of cancer‐associated muscle wasting.

The Akt/mTORC1 signalling pathway plays an important role in adult muscle homeostasis. This pathway has been observed to be remarkably inhibited in the context of cancer cachexia–related ER stress in this study and others [16, 25]. Although the molecular mechanism behind reduced Akt/mTOR activity has not been well understood, this study demonstrates that NatC links ER stress and Akt/mTOR through CTSK‐mediated IRS1 degradation. Deletion of NatC partially restored Akt/mTOR signalling and resulted in inhibition of loss of muscle mass and function. Intervention on Akt/mTOR as well as on CTSK has been reported to prevent muscle wasting in a mouse model of cancer cachexia [16, 25]. Attenuated Akt/mTOR signalling and consequent decreased protein synthesis and upregulated protein degradation via UPS and ALS were observed in LCC and colon cancer models. Activation of mTOR in inducible Akt1 transgenic mice rescued these changes and completely reversed the 15%–20% loss of muscle mass and force [16]. One mechanism underlying impaired Akt/mTOR signalling is CTSK. Expression of the cathepsin family, including CTSK, is upregulated in muscle atrophy of various aetiologies. CTSK is an endopeptidase, yet it is also capable of directly degrading covalently bound substrates. IRS1 is one such target, and deletion of CTSK preserved IGF1/IRS1 signalling including Akt/mTOR pathway and prevented muscle atrophy [25]. Our findings of NatC‐mediated stabilization of CTSK under ER stress are consistent with these results and explain the detailed mechanism underlying the inhibition of Akt/mTOR signalling in muscle wasting due to cachexia.

The NatC protein is a member of the Nat family and is responsible for Nt‐acetylation in specific proteins that begin with methionine, followed by a hydrophobic or amphipathic amino acid (ML−, MF−, MI− and MW−) [9]. In general, Nt‐acetylation affects protein stability, protein turnover via proteasome, protein–protein interaction, protein subcellular localization and protein folding [10]. Nt‐acetylated proteins could be recognized as degrons by the N‐end rule pathways [36], leading to low levels of protein expression. In contrast, Nt‐acetylation could also promote protein stability, and some unacetylated N‐terminus could be recognized as degrons [37]. This evidence suggests that Nt‐acetylation affects protein half‐life in a context‐dependent manner. In this study, knockdown of Naa35 reduced CTSK protein expression without affecting its mRNA level under ER stress from tunicamycin treatment (Figure 6A), indicating that posttranslational modification of Nt‐acetylation supports CTSK stability and protein expression level in cancer cachexia muscles.

The NatC components were upregulated in drug‐induced ER stress and cancer cachexia. The UPR is initiated by three ER stress sensors, PERK, ATF6 and IRE1α. Experiments with selective inhibitors and activators of these sensors identified ATF6 as responsible for upregulating NatC transcription. Persistent ATF6 activation induces the transcription of CHOP that is involved in apoptosis [38]. Skeletal muscle from LLC‐bearing cachexia model mice exhibited the upregulation of CHOP protein (Figure 1F). This result supports the evidence of ATF6 pathway activation in cancer cachexia and also suggests that, although we have not evaluated it, apoptosis may contribute to muscle atrophy along with impaired Akt/mTOR signaling.

There are several limitations of our current study. We unbiasedly identified NatC as a regulator of ER stress–mediated muscle atrophy and confirmed the CTSK protein expression level was regulated downstream of NatC. Although the molecular mechanism is Nt‐acetylation, we have not evaluated the Nt‐acetylation of CTSK. Direct evidence of NatC‐mediated acetylation of CTSK further supports the hypothesis that increased CTSK is involved in the impairment of Akt/mTOR signalling in muscle wasting in cancer cachexia. In general, NatC broadly acetylates the N‐terminus of proteins with of ML−, MF−, MI− and MW−. Therefore, CTSK is not the only target of NatC in ER stress and other proteins. Nt‐acetylation may also regulate muscle atrophy in cancer cachexia. Regarding an ATF6 inhibitor, we used AEBSF because it is widely employed to block the site‐1/site‐2 protease–dependent cleavage of ATF6 during ER stress [21]. However, AEBSF is a broad serine protease inhibitor and is not specific to ATF6, raising the possibility of off‐target effects. To address this concern, we conducted a complementary experiment using AA147, a well‐characterized ATF6 activator. Activation of ATF6 by AA147 induced phenotypic changes opposite to those produced by AEBSF treatment, consistent with the expected role of ATF6 signalling. These findings strengthen our interpretation that modulation of ATF6 activity contributes to the observed effects, despite the non‐specific nature of AEBSF. Future studies employing more selective genetic approaches, such as ATF6 knockdown or targeted inhibition of site‐1/site‐2 proteases, will definitively establish the causal role of ATF6 in NatC‐related muscle pathology. To evaluate muscle function in the present study, we employed the whole‐limb grip‐strength test, a widely used non‐invasive measure of in vivo neuromuscular performance. The gold‐standard approach for quantifying intrinsic muscle contractile force is the in situ force measurement of isolated muscles such as the TA. However, these assays require invasive surgical procedures and specialized equipment, making them not feasible within the scope of this study. Importantly, the validity of grip strength as an indicator of skeletal muscle dysfunction has been supported by previous work. Takeshita et al. demonstrated that reduced forelimb grip strength in mdx mice occurred in parallel with impaired twitch and maximal tetanic force of the TA muscle measured in situ, indicating that grip strength reflects systemic muscle dysfunction in this model [39]. In addition, grip strength has been widely recognized as a standard, non‐invasive readout of limb muscle function in rodent studies [40]. Because our experimental intervention specifically targeted the hindlimb muscles (including the TA) and did not involve the forelimbs, we consider that changes detected by grip strength reasonably approximate functional alterations in hindlimb muscle performance in this context. Nevertheless, future studies incorporating direct in situ force measurements will definitively determine the impact of NatC on intrinsic muscle contractile capacity.

In conclusion, NatC was identified as a critical determinant of ER stress–mediated muscle atrophy in cancer cachexia by in vitro genome‐wide CRISPR library screening. Among UPR branches, ATF6 was responsible for NatC upregulation and CTSK stability through Nt‐acetylation and consequent IRS1 degradation, linking ER stress and impaired Akt/mTOR signalling. Knockdown of Naa35 alleviated the loss of muscle mass and function in cancer‐bearing mice, shedding light on NatC as a therapeutic target for cancer cachexia muscle wasting.

## Funding

This research was funded by a JSPS Grant‐in‐Aid Scientific Research Grant (22H03071 and 25K02651) and the Nakatomi Foundation.

## Ethics Statement

This study was approved by the Animal Care and Use Committee of Kyoto Prefectural University of Medicine (Approval Numbers M2021‐565, M2022‐116 and M2023‐104).

## Conflicts of Interest

The authors declare no conflicts of interest.

## Supporting information


**Figure S1:** NatC is an ER stress–resistant regulator in myocytes. (A) RRA score of gRNAs that preserved cell growth under ER stress in the MAGeCK analysis of the CRISPR library screen. (B) Flow cytometry analysis to evaluate the effect of Naa35 on cell growth under treatment with 4 nM thapsigargin (TG) for 7 days. Data are expressed as mean ± SD. The *p* values were determined by unpaired *t*‐test. (C) The effect of Naa35 on the cell growth of Neuro2A cells under treatment of 160 ng/mL tunicamycin (TM) for 7 days. Data are expressed as mean ± SD. The *p* values were determined by unpaired *t*‐test.
**Figure S2:** NatC knockout is not protective against oxidative stress and genotoxicity. (A) Staining for ROS with CellROX Green after exposure of C2C12 to 1 mM H_2_O_2_ or 160 ng/mL tunicamycin (TM) with or without N‐acetyl‐L‐cysteine (NAC) for 1 h and flow cytometry analysis of these cells. Scale bar, 100 μm. (B) Flow cytometry analysis of cell growth after exposure of C2C12 cells to 1 mM of H_2_O_2_ or 1 μM doxorubicin (DOX) for 3 days.
**Figure S3:** ER stress–mediated myotube atrophy was reproduced by the conditioned medium of C26 colon carcinoma cells. (A) Immunofluorescence staining for desmin and quantification of diameter in differentiated myotubes treated with tunicamycin (TM) or 50% C26 conditioned medium (C26‐CM) for 72 h. Scale bar, 100 μm. Data are expressed as median. The *p* values were determined by one‐way ANOVA with Tukey's multiple comparison test. (B) Gene expressions of ER stress markers (Bip, Xbp1s, CHOP, ATF4 and ATF6) and atrophy‐related genes (Fbxo31, MUSA1, MuRF1 and MAFbx1) in myotubes after 50% C26‐CM treatment for 72 h. Gene expression levels were quantified using the ΔΔCt method and normalized to GAPDH. Relative expressions are shown as fold change compared with the control group. Data are expressed as mean ± SD. The *p* values were determined by unpaired *t*‐test. (C) Immunoblot of myotubes after treatment with 50% C26‐CM for 72 h. (D) Immunostaining images of myotubes and diameter after 50% C26‐CM treatment for 72 h. Scale bar, 100 μm. Data are expressed as median. The *p* values were determined by one‐way ANOVA with Tukey's multiple comparison test. (E) qPCR analysis of atrogenes (MuRF1 and MAFbx1). Gene expression levels were quantified using the ΔΔCt method and normalized to GAPDH. Relative expressions are shown as fold change compared with the control si‐NC group. Data are expressed as mean ± SD. The *p* values were determined by one‐way ANOVA with Tukey's multiple comparison test.
**Figure S4:** NatC silencing has no effect on steroid‐induced myotube atrophy. (A) qPCR analysis of ER stress markers (Bip, Xbp1s, CHOP, ATF4 and ATF6) and NatC (Naa35, Naa38 and Naa30) after treatment with 10 μM dexamethasone (DEX) for 48 h. (B) Immunostaining of myotubes for desmin and quantification of myotube diameter after treatment with 10 μM DEX for 48 h. Scale bar, 100 μm. Data are expressed as median. The *p* values were determined by one‐way ANOVA with Tukey's multiple comparison test.
**Figure S5:** ATF6 is involved in thapsigargin‐induced NatC expression. Gene expression levels of NatC components, Naa35, Naa38 and Naa30 in C2C12 cells treated with 1 μM thapsigargin (TG) and inhibitors, 1 μM GSK2606414, 3 μM Kira6 and 400 μM AEBSF for 15 h. Data are expressed as mean ± SD. The *p* values were determined by one‐way ANOVA with Tukey's multiple comparison test.
**Figure S6:** Cathepsin K is the target of NatC and involved in the myotube atrophy. (A) C2C12 cells were transfected with si‐Nc or si‐Naa35 and 2 days later treated with 160 ng/mL tunicamycin (TM) or 1 μM thapsigargin (TG) for 15 h. qPCR analysis of Bip, Xbp1s and CHOP in whole‐cell lysates of cells. Data are expressed as mean ± SD. The *p* values were determined by one‐way ANOVA with Tukey's multiple comparison test. (B) Amino acid sequence of Cathepsin K. (C) Immunostaining images of myotubes and diameter after 50% LLC‐CM treatment for 48 h. Scale bar, 100 μm. Data are expressed as median. The *p* values were determined by one‐way ANOVA with Tukey's multiple comparison test. (D) qPCR analysis of CTSK and atrogenes (MuRF1 and MAFbx1). Gene expression levels were quantified using the ΔΔCt method and normalized to GAPDH. Relative expressions are shown as fold change compared with the control si‐NC group. Data are expressed as mean ± SD. The *p* values were determined by one‐way ANOVA with Tukey's multiple comparison test. (E) Immunoblot analysis of IRS1, p‐S6, S6, p‐Akt, Akt and β‐actin in whole lysates of C2C12 myotube treated with siRNA targeting CTSK.
**Table S1:** qPCR primers.
