# Supplementary material for: Inhibition of N‐Terminal Acetyltransferase C Mitigates Endoplasmic Reticulum Stress–Mediated Muscle Atrophy in Cancer Cachexia
Source: J Cachexia Sarcopenia Muscle. 2026 Mar 19;17(2):e70249. doi: 10.1002/jcsm.70249 (PMC13140329; doi:10.1002/jcsm.70249)
Supplement: Supplementary file 1 — Figure S1: NatC is an ER stress–resistant regulator in myocytes. (A) RRA score of gRNAs that preserved cell growth under ER stress in the MAGeCK analysis of the CRISPR library screen. (B) Flow cytometry analysis to evaluate the effect of Naa35 on cell growth under treatment with 4 nM thapsigargin (TG) for 7 days. Data are expressed as mean ± SD. The p values were determined by unpaired t‐test. (C) The effect of Naa35 on the cell growth of Neuro2A cells under treatment of 160 ng/mL tunicamycin (TM) for 7 days. Data are expressed as mean ± SD. The p values were determined by unpaired t‐test. Figure S2: NatC knockout is not protective against oxidative stress and genotoxicity. (A) Staining for ROS with CellROX Green after exposure of C2C12 to 1 mM H2O2 or 160 ng/mL tunicamycin (TM) with or without N‐acetyl‐L‐cysteine (NAC) for 1 h and flow cytometry analysis of these cells. Scale bar, 100 μm. (B) Flow cytometry analysis of cell growth after exposure of C2C12 cells to 1 mM of H2O2 or 1 μM doxorubicin (DOX) for 3 days. Figure S3: ER stress–mediated myotube atrophy was reproduced by the conditioned medium of C26 colon carcinoma cells. (A) Immunofluorescence staining for desmin and quantification of diameter in differentiated myotubes treated with tunicamycin (TM) or 50% C26 conditioned medium (C26‐CM) for 72 h. Scale bar, 100 μm. Data are expressed as median. The p values were determined by one‐way ANOVA with Tukey's multiple comparison test. (B) Gene expressions of ER stress markers (Bip, Xbp1s, CHOP, ATF4 and ATF6) and atrophy‐related genes (Fbxo31, MUSA1, MuRF1 and MAFbx1) in myotubes after 50% C26‐CM treatment for 72 h. Gene expression levels were quantified using the ΔΔCt method and normalized to GAPDH. Relative expressions are shown as fold change compared with the control group. Data are expressed as mean ± SD. The p values were determined by unpaired t‐test. (C) Immunoblot of myotubes after treatment with 50% C26‐CM for 72 h. (D) Immunostaining images of myotubes [file JCSM-17-e70249-s001.docx]

**Inhibition of N-terminal acetyltransferase C mitigates ER stress-mediated muscle atrophy in cancer cachexia**

Yusaku Kaneko^1^, Tomohiro Hino^1^, Shunta Taminishi^1^, Yayoi Matoba^2^, Daisuke Motooka^3,4^, Atsushi Hoshino^1^*, Satoaki Matoba^1^

^1^ Department of Cardiovascular Medicine, Graduate School of Medical Science, Kyoto Prefectural University of Medicine, Kyoto 602-8566, Japan.

^2^ Department of Nephrology, Graduate School of Medical Science, Kyoto Prefectural University of Medicine, Kyoto 602-8566, Japan.

^3^ Department of Infection Metagenomics, Research Institute for Microbial Diseases, Osaka University, Osaka, Japan.

^4^ Integrated Frontier Research for Medical Science Division, Institute for Open and Transdisciplinary Research Initiatives (OTRI), Osaka University, Osaka 565-0871, Japan.

*Correspondence: Atsushi Hoshino (a-hoshi@koto.kpu-m.ac.jp)


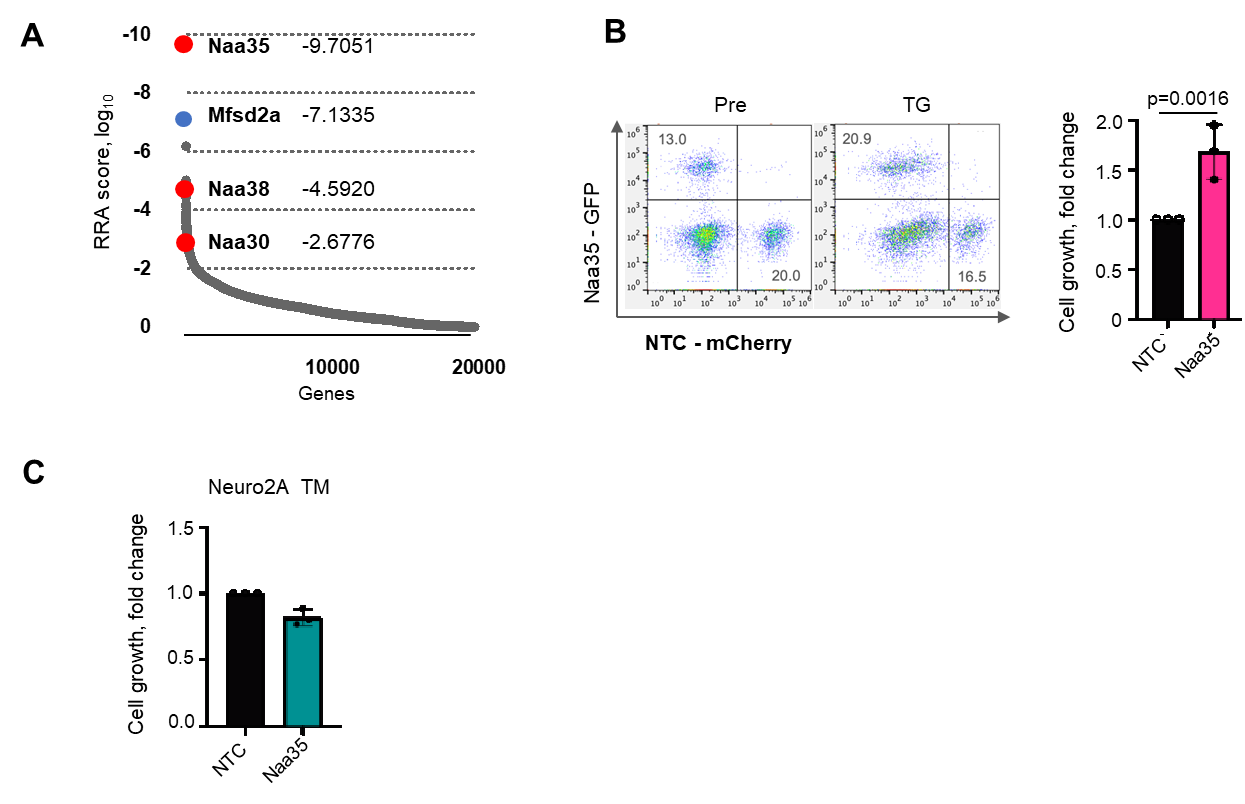


Figure S1 **NatC is an ER stress-resistant regulator in myocytes.** (A) RRA score of gRNAs that preserved cell growth under ER stress in the MAGeCK analysis of the CRISPR library screen. (B) Flow cytometry analysis to evaluate the effect of Naa35 on cell growth under treatment with 4 nM thapsigargin (TG) for 7 days. Data are expressed as mean ± SD. *P* values were determined by unpaired *t* test. (C) The effect of Naa35 on the cell growth of Neuro2A cells under treatment of 160 ng/mL tunicamycin (TM) for 7 days. Data are expressed as mean ± SD. *P* values were determined by unpaired *t* test.


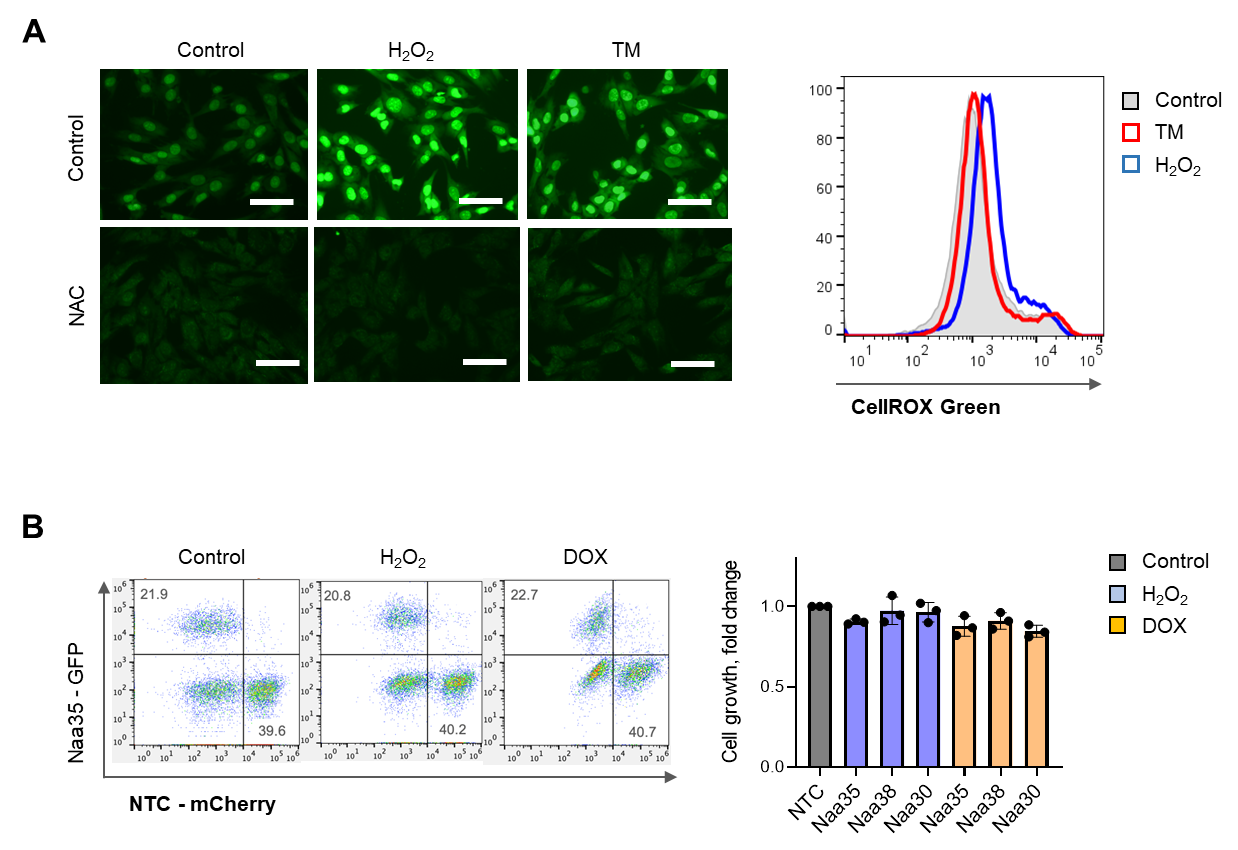


Figure S2 **NatC knockout is not protective against oxidative stress and genotoxicity.** (A) Staining for ROS with CellROX Green after exposure of C2C12 to 1 mM H_2_O_2_ or 160 ng/mL tunicamycin (TM) with or without N-acetyl-L-cysteine (NAC) for 1 hour and flow cytometry analysis of these cells. Scale bar, 100 μm. (B) Flow cytometry analysis of cell growth after exposure of C2C12 cells to 1 mM of H_2_O_2_ or 1 µM doxorubicin (DOX) for 3 days.


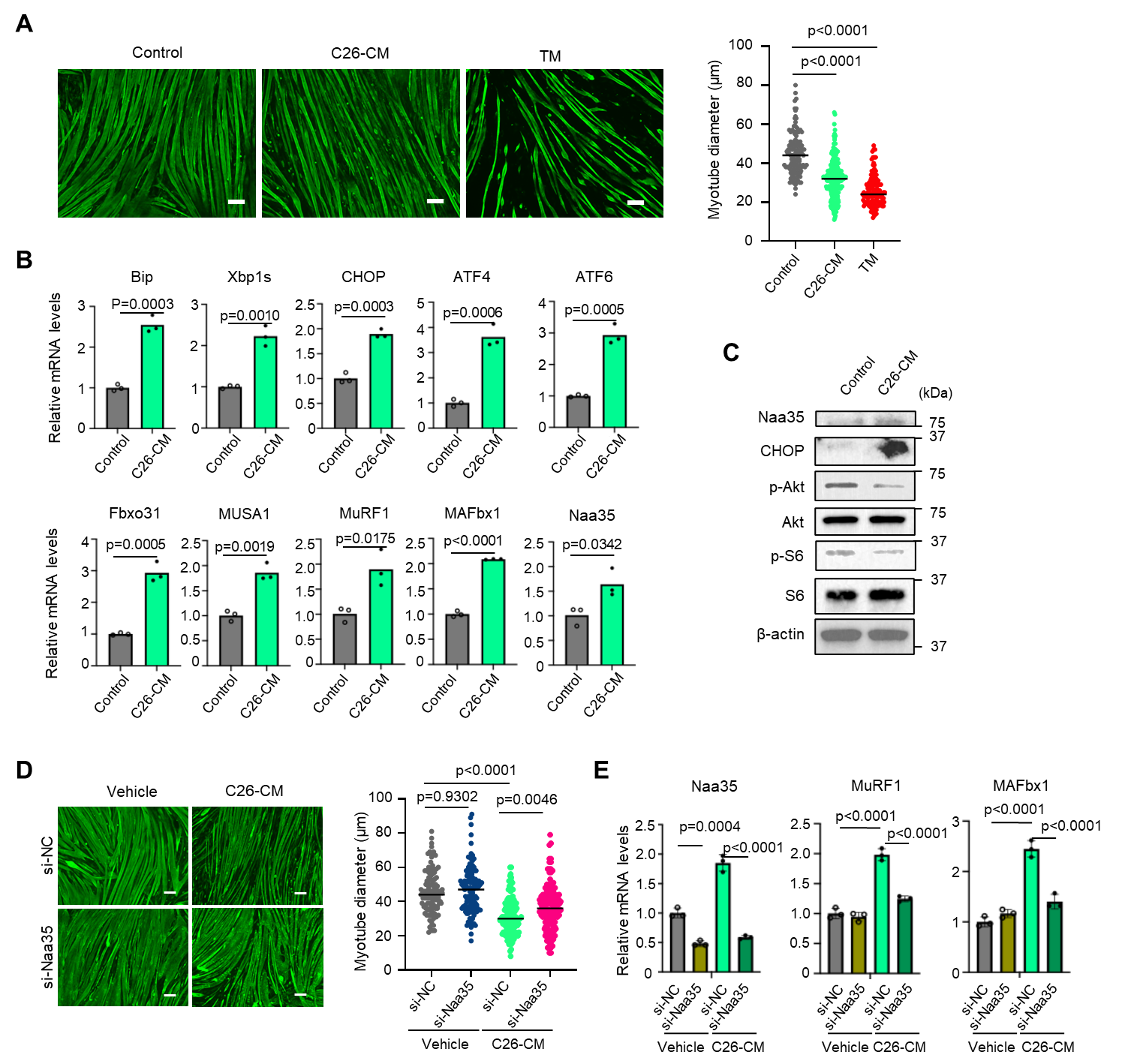


Figure S3 **ER stress-mediated myotube atrophy was reproduced by the conditioned medium of C26 colon carcinoma cells.** (A) Immunofluorescence staining for desmin and quantification of diameter in differentiated myotubes treated with tunicamycin (TM) or 50% C26 conditioned medium (C26-CM) for 72 hours. Scale bar, 100μm. Data are expressed as median. *P* values were determined by one-way ANOVA with Tukey’s multiple comparisons test. (B) Gene expressions of ER stress markers (Bip, Xbp1s, CHOP, ATF4, ATF6) and atrophy-related genes (Fbxo31, MUSA1, MuRF1, MAFbx1) in myotubes after 50% C26-CM treatment for 72 hours. Gene expression levels were quantified using the ΔΔCt method and normalized to **GAPDH**. Relative expressions are shown as fold-change compared with the control group. Data are expressed as mean ± SD. *P* values were determined by unpaired *t* test. (C) Immunoblot of myotubes after treatment with 50% C26-CM for 72 hours. (D) Immunostaining images of myotubes and diameter after 50% C26-CM treatment for 72 hours. Scale bar, 100μm. Data are expressed as median. *P* values were determined by one-way ANOVA with Tukey’s multiple comparisons test. (E) qPCR analysis of atrogenes (MuRF1, MAFbx1). Gene expression levels were quantified using the ΔΔCt method and normalized to **GAPDH**. Relative expressions are shown as fold-change compared with the control si-NC group. Data are expressed as mean ± SD. *P* values were determined by one-way ANOVA with Tukey’s multiple comparisons test.


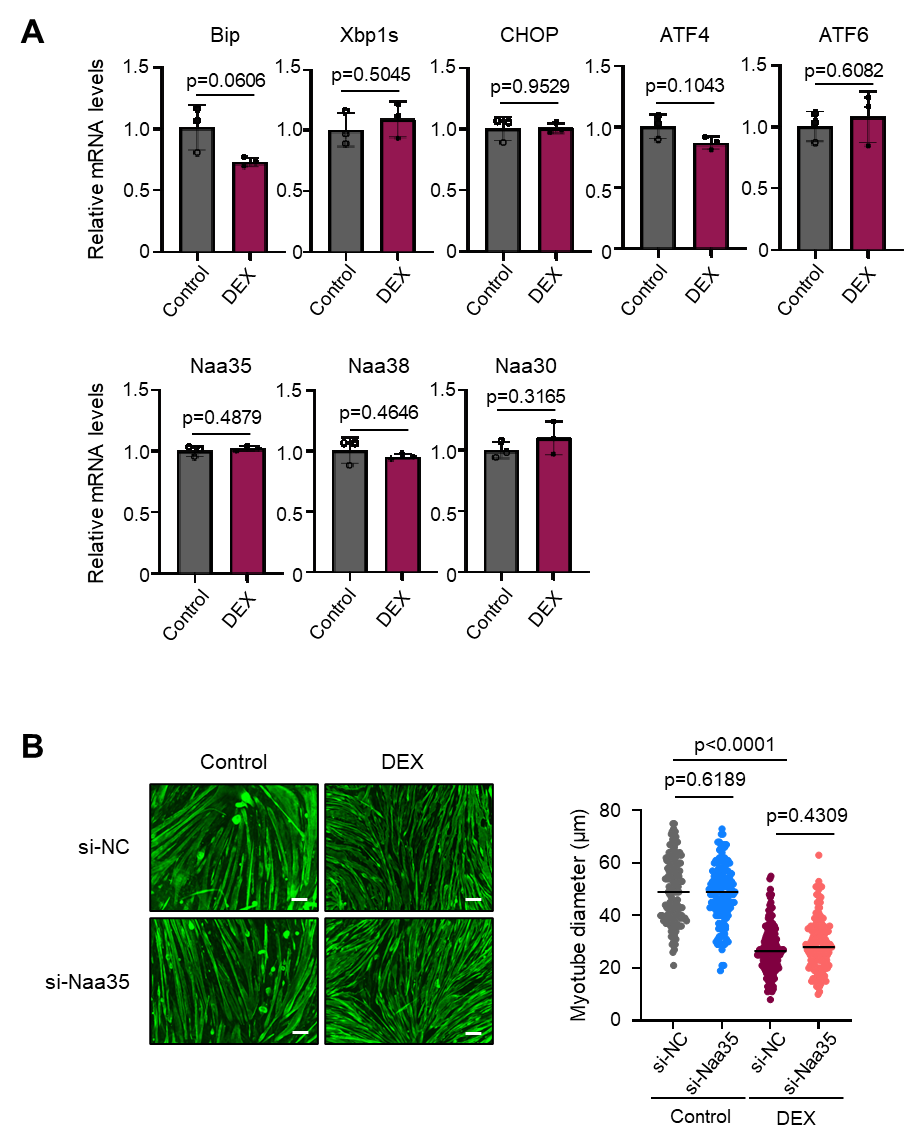


Figure S4 **NatC silencing has no effect on steroid-induced myotube atrophy.** (A) qPCR analysis of ER stress markers (Bip, Xbp1s, CHOP, ATF4, and ATF6) and NatC (Naa35, Naa38, and Naa30) after treatment with 10 µM dexamethasone (DEX) for 48 hours. (B) Immunostaining of myotubes for desmin and quantification of myotube diameter after treatment with 10 µM DEX for 48 hours. Scale bar, 100μm. Data are expressed as median. *P* values were determined by one-way ANOVA with Tukey’s multiple comparisons test.


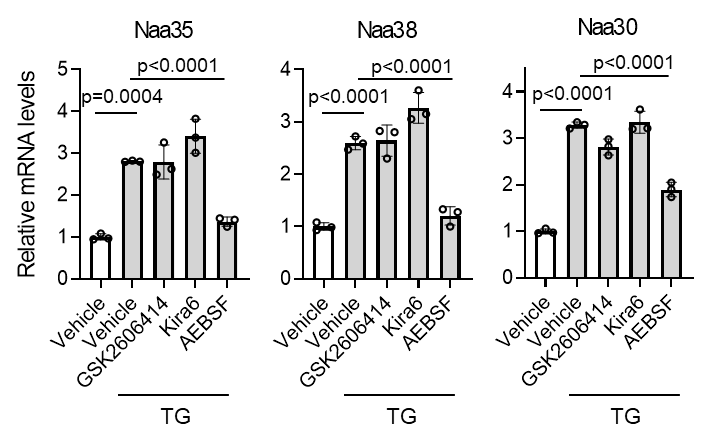


Figure S5 **ATF6 is involved in thapsigargin-induced NatC expression.** Gene expression levels of NatC components, Naa35, Naa38, and Naa30 in C2C12 cells treated with 1 µM thapsigargin (TG) and inhibitors, 1 µM GSK2606414, 3 µM Kira6, and 400 µM AEBSF for 15 hours. Data are expressed as mean ± SD. *P* values were determined by one-way ANOVA with Tukey’s multiple comparisons test.


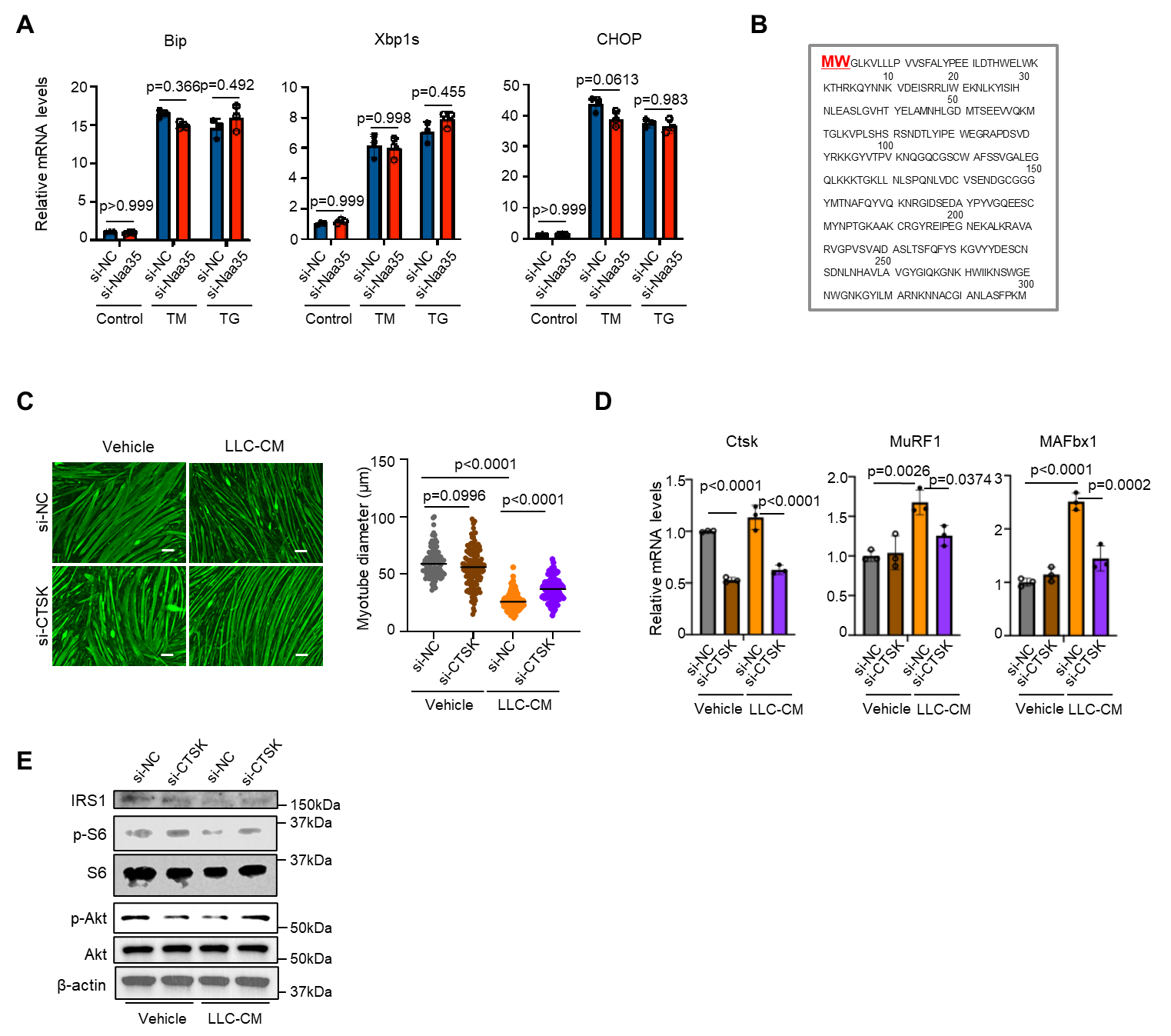


Figure S6 **Cathepsin K is the target of NatC and involved in the myotube atrophy.** (A) C2C12 cells were transfected with si-Nc or si-Naa35 and 2 days later treated with 160 ng/mL tunicamycin (TM) or 1 µM thapsigargin (TG) for 15 hours. qPCR analysis of Bip, Xbp1s, CHOP in whole-cell lysates of cells. Gene expression levels were quantified using the ΔΔCt method and normalized to GAPDH. Relative expressions are shown as fold-change compared with the control si-NC group. Data are expressed as mean ± SD. *P* values were determined by one-way ANOVA with Tukey’s multiple comparisons test. (B) Amino acid sequence of Cathepsin K. (C) Immunostaining images of myotubes and diameter after 50% LLC-CM treatment for 48 hours. Scale bar, 100μm. Data are expressed as median. *P* values were determined by one-way ANOVA with Tukey’s multiple comparisons test. (D) qPCR analysis of CTSK and atrogenes (MuRF1, MAFbx1). Gene expression levels were quantified using the ΔΔCt method and normalized to GAPDH. Relative expressions are shown as fold-change compared with the control si-NC group. Data are expressed as mean ± SD. *P* values were determined by one-way ANOVA with Tukey’s multiple comparisons test. (E) Immunoblot analysis of IRS1, p-S6, S6, p-Akt, Akt, β-actin in whole lysates of C2C12 myotube treated with siRNA targeting CTSK.

Table S1 qPCR primers

| Name | forward | reverse |
| --- | --- | --- |
| Naa35 | 5’-AGCTACTGTAGACGACGATGC-3’ | 5’-AACTTCAGCTCTCGACAAGCA-3’ |
| Naa38 | 5’-GGGCCGACCATGCTACTAC-3’ | 5’-CTTGTTGAGCAATGCCTCTAGTT-3’ |
| Naa30 | 5’-TGCCATGGTTGAAGGAGACTGTG-3’ | 5’-CAGCCTCTTATCTCGAACAAAACC-3’ |
| Bip | 5’-ACTTGGGGACCACCTATTCCT-3’ | 5’-ATCGCCAATCAGACGCTCC-3’ |
| Xbp1s | 5’-GAGTCCGCAGCAGGTG-3’ | 5’-GTGTCAGAGTCCATGGGA-3’ |
| CHOP | 5’-CTGGAAGCCTGGTATGAGGAT-3’ | 5’-CAGGGTCAAGAGTAGTGAAGGT-3’ |
| ATF4 | 5’-ATGGCGCTCTTCACGAAATC-3’ | 5’-ACTGGTCGAAGGGGTCATCAA-3’ |
| ATF6 | 5’-GACTCACCCATCCGAGTTGTG-3’ | 5’-CTCCCAGTCTTCATCTGGTCC-3’ |
| MuRF1 | 5’-TGATTCCTGATGGAAACGCTATGG-3’ | 5’-ATTCGCAGCCTGGAAGATGTC-3’ |
| MAFbx1 | 5’-GACAAAGGGCAGCTGGATTGG-3’ | 5’-TCAGTGCCCTTCCAGGAGAGA-3’ |
| Fbxo31 | 5’-GTATGGCGTTTGTGAGAACC-3’ | 5’-AGCCCCAAAATGTGTCTGTA-3’ |
| MUSA1 | 5’-TCGTGGAATGGTAATCTTGC-3’ | 5’-CCTCCCGTTTCTCTATCACG-3’ |
| CTSK | 5’-GAAGAAGACTCACCAGAAGCAG-3’ | 5’-TCCAGGTTATGGGCAGAGATT-3’ |
